# Characterization of a panel of Vietnamese rice varieties using DArT and SNP markers for association mapping purposes

**DOI:** 10.1186/s12870-014-0371-7

**Published:** 2014-12-19

**Authors:** Nhung Thi Phuong Phung, Chung Duc Mai, Pierre Mournet, Julien Frouin, Gaëtan Droc, Nhung Kim Ta, Stefan Jouannic, Loan Thi Lê, Vinh Nang Do, Pascal Gantet, Brigitte Courtois

**Affiliations:** Agricultural Genetics Institute, National Key Laboratory for Plant Cell Biotechnology, LMI RICE, Hanoi, Vietnam; Cirad, UMR-AGAP, 34398 Montpellier, France; IRD, UMR-DIADE, LMI RICE, Hanoi, Vietnam; Université Montpellier 2, UMR DIADE, 34095 Montpellier, France; University of Science and Technology of Hanoi, LMI RICE, Hanoi, Vietnam; Plant Resource Center, Hanoi, Vietnam

**Keywords:** DArT markers, SNP, Genetic diversity, Linkage disequilibrium, Rice, Vietnam

## Abstract

**Background:**

The development of genome-wide association studies (GWAS) in crops has made it possible to mine interesting alleles hidden in gene bank resources. However, only a small fraction of the rice genetic diversity of any given country has been exploited in the studies with worldwide sampling conducted to date. This study presents the development of a panel of rice varieties from Vietnam for GWAS purposes.

**Results:**

The panel, initially composed of 270 accessions, was characterized for simple agronomic traits (maturity class, grain shape and endosperm type) commonly used to classify rice varieties. We first genotyped the panel using Diversity Array Technology (DArT) markers. We analyzed the panel structure, identified two subpanels corresponding to the *indica* and *japonica* sub-species and selected 182 non-redundant accessions. However, the number of usable DArT markers (241 for an initial library of 6444 clones) was too small for GWAS purposes. Therefore, we characterized the panel of 182 accessions with 25,971 markers using genotyping by sequencing. The same *indica* and *japonica* subpanels were identified. The *indica* subpanel was further divided into six populations (I1 to I6) using a model-based approach. The *japonica* subpanel, which was more highly differentiated, was divided into 4 populations (J1 to J4), including a temperate type (J2). Passport data and phenotypic traits were used to characterize these populations. Some populations were exclusively composed of glutinous types (I3 and J2). Some of the upland rice varieties appeared to belong to *indica* populations, which is uncommon in this region of the world. Linkage disequilibrium decayed faster in the *indica* subpanel (r^2^ below 0.2 at 101 kb) than in the *japonica* subpanel (r^2^ below 0.2 at 425 kb), likely because of the strongest differentiation of the *japonica* subpanel. A matrix adapted for GWAS was built by eliminating the markers with a minor allele frequency below 5% and imputing the missing data. This matrix contained 21,814 markers. A GWAS was conducted on time to flowering to prove the utility of this panel.

**Conclusions:**

This publicly available panel constitutes an important resource giving access to original allelic diversity. It will be used for GWAS on root and panicle traits.

**Electronic supplementary material:**

The online version of this article (doi:10.1186/s12870-014-0371-7) contains supplementary material, which is available to authorized users.

## Background

Rice is the major crop in Vietnam, occupying 70% of the total agricultural area [1]. Rice is cultivated in all types of ecosystems (irrigated, rainfed lowland, flood-prone, upland and mangrove) because of the large diversity of landscapes. However, the irrigated ecosystem, located primarily in the Mekong River delta in the South and in the Red River delta in the North, accounts by itself for approximately half of the harvested rice area, with two to three rice crops per year [2]. North Vietnam is said to lie within the center of genetic diversity of Asian cultivated rice and, as such, the rice diversity in this area is high [3]. However, in the less favorable ecosystems, rice is progressively abandoned as unprofitable. To limit the erosion of genetic resources, which is linked to crop diversification, and the disappearance of traditional varieties that is a particularly threat to upland rice, several rounds of collection of traditional varieties have been undertaken throughout Vietnam since 1987. Local genetic resources are conserved in Vietnamese gene banks that are members of a national network [4]. However, little genetic characterization of these genetic resources has been performed and most of the studies that are available were conducted on limited sets of accessions, using isozymes [5,6], restriction fragment length polymorphisms [3] and, more recently, microsatellite markers [7]. Genetic analyses are necessary to add value to gene bank collections, as shown by Tanksley and McCouch [8]. These analyses help to improve our understanding of rice diversity, enabling more effective conservation and use of that diversity in breeding programs, thereby justifying the sustained investment of resources into gene bank collections. With the development of genome-wide association studies (GWAS) in crops [9], there has been a renewed interest in genetic resources, with the objective of mining interesting alleles hidden in gene bank resources. The recent discoveries of agronomically important genes present in traditional rice varieties that are absent in the reference variety *Nipponbare*, e.g. *SUB1* for submergence tolerance or *PSTOL1* for phosphate uptake, illustrate the usefulness of this approach [10,11].

GWAS is a method used to dissect the genetic basis of the variation in complex quantitative traits by establishing statistical links between phenotypes and genotypes [12]. The two major advantages of GWAS over classical QTL detection in mapping populations are that GWAS can be conducted directly on panels of varieties without having to develop specific mapping populations and that GWAS enable the exploration of the large diversity of alleles present in genetic resources. GWAS rely on the linkage disequilibrium (LD) that exists in a population or species [13]. With LD spanning a short distance, the resolution of association mapping will be excellent, but the number of markers needed to cover the genome is high. Conversely with LD spanning a longer distance, the resolution will be poor, but the marker density does not need to be high. The rate of LD decay with physical distance depends on the panel and, within a given panel, also varies depending on the chromosomal segment under consideration. LD therefore has to be evaluated in depth to determine whether the tagging of the genome is sufficient for GWAS purposes. In rice, previous studies have given an overall value of LD decay in *Oryza sativa* in the range of 75 to more than 500 kb, depending on the population considered [14].

The GWAS approach carries some drawbacks. Population structure is a major limitation to successful association studies in any organism because it may induce high rates of false positives in the analyses, although this rate can be controlled by statistical methods using elements describing this structure (percentages of admixture and/or kinship matrices) as cofactors into the analyses [15,16]. A good understanding of population structure is therefore of primary importance before conducting GWAS. *O. sativa* is a highly structured species with two major sub-species, *indica* and *japonica*, that diverged long ago [17,18]. In addition to this bipolar structure, a finer structure has been recognized in five groups. The *indica* and *aus* groups are part of the *indica* sub-species, from which the tiny *aswina* and *rayada* groups are sometimes individualized [19]. The *aromatic* and *japonica* groups are part of the large *japonica* sub-species, the latter further subdivided into *tropical* and *temperate* components [20]. Therefore, accurate control of the genetic structure of the panel used for association studies is particularly needed in the case of rice and a within-sub-species or within-varietal group analysis can be useful as was done for the first GWAS conducted in rice [21,22].

Because of the limited LD of natural populations, GWAS requires a high marker density, which is only possible today because of the developments in high-throughput genotyping and sequencing. An initial set of 35 Vietnamese rice varieties has recently been fully sequenced [23], but this sample is not large enough to enable reliable association studies.

Markers adapted for high-throughput genotyping are available. DArT (Diversity Array Technology) markers were developed by Jaccoud et al. [24] to enable whole genome profiling of crops without the need for sequence information. The first step of marker development involves the creation of a library of genomic fragments using restriction enzymes to digest DNA and reduce genome complexity. Fragments selected from the library are spotted on a glass slide using a microarray platform. The target DNA is treated in the same way as the DNA used to constitute the library. It is digested with the same enzymes, and the fragments are hybridized on a chip to reveal the presence/absence of certain sequences. Because of the presence/absence allele calling, DArT markers are dominant markers. DArT markers have been rarely utilized in rice [24,25]. For other species, these markers have proved efficient at displaying accurate patterns of genetic diversity in homozygous crops [26] as well as highly heterozygous crops [27,28]. DArT markers have also been used to build genetic maps [29] and to genotype association mapping panels [26].

Single nucleotide polymorphisms (SNPs) are single base substitutions. The advantage of SNPs as markers is that they have a very high density in the genome, approximately 1.6 to 1.7 SNPs/kb in rice [30,31]. To genotype SNPs, a recently developed method, genotyping by sequencing (GBS), is becoming increasingly popular [32]. As for DArT markers, the genomic DNA is digested with restriction enzymes adapted to the targeted marker density. Enzyme-specific adapters tagged with different barcodes are then ligated to the restriction fragments and the restricted fragments which are sequenced using Illumina short-read sequencing. The sequences are aligned to the reference species genome and SNPs are identified in the sequences. This method has been described in detail by Elshire et al. [33] and has already been used for all possible applications in rice: genetic diversity, genetic mapping, association mapping and genomic selection [34-36].

This paper presents the results of a genetic characterization of a set of traditional Vietnamese accessions, first with DArT markers and then with SNP markers genotyped at high density. Population structure and LD decay were finely analyzed at different levels of organization to assess to what extent the panel is appropriate for association mapping studies and will eventually enable the identification of new agronomically relevant alleles. A GWAS was then conducted on a simple trait to reveal what types of results can be expected from this panel.

## Methods

### Materials

The initial collection analyzed was composed of 270 varieties (Additional file 1: Table S1). The majority of the accessions (214) were traditional varieties provided by the Plant Resource Center (Hanoi, Vietnam) that originated from different districts of Vietnam and diverse rice ecosystems (Additional file 1: Table S1). Some of the accessions (32) were chosen from a core collection representing the varietal group diversity of *Oryza sativa* for which the enzymatic group is known [37]. This set is hereafter referred to as the "reference set". One accession from *O. glaberrima* provided by the Institut de recherche pour le développement (Montpellier, France) was added as an outgroup. The remaining accessions (23) were well known varieties from Asia provided by the Agronomical Genetics Institute (Hanoi, Vietnam). Information on the country of origin, the district for Vietnamese varieties, the varietal type (traditional or improved), and the ecosystem (irrigated, rainfed lowland, upland, or mangrove) are given in (Additional file 1: Table S1) for the Vietnamese accessions and in (Additional file 1: Table S2) for the two other sets.

### DNA extraction

DNA was extracted from one plant per accession using the CTAB method [38]. The DNA concentration was visually checked in reference to well quantified samples after agarose gel electrophoresis and ethidium bromide staining, and all samples were diluted to 100 ng/μl.

### Genotyping with DArT markers

A preliminary step to use DArT markers is to develop a library of DNA fragments. A library of 6144 clones was built from 25 varieties, including 10 indica accessions and 15 temperate and tropical japonicas by the DArT platform of Cirad (Additional file 1: Table S3). The method to build the library was similar to that described in detail by Jaccoud et al. [24] and Risterucci et al. [28]. Only the overall strategy and changes to the standard protocol are reported here. Briefly, each sample was digested with two restriction enzymes, the rare cutter PstI (6 bp recognition site) and the frequent cutter TaqI (4 bp recognition site). The restriction product was then ligated to a PstI adapter and amplified by PCR using a primer complementary to the adapter sequence. The amplification products were cloned into a pGEM-T easy vector that was transformed into *Escherichia coli* to generate the library. Within the library, each colony contains one of the PCR-amplified DNA fragments of the genomic representations [24]. The 6144 amplicons of the rice library were spotted on amino-silane-coated microarray slides using a microarrayer.

The target DNA samples were prepared using the same complexity-reduction method as the library DNA and labeled with a Cy3/Cy5 fluorescent label, as described by Risterucci et al. [28]. After denaturing, each sample was hybridized onto a slide. The slides were scanned using a fluorescent microarray scanner. For each slide, the scores of the 6144 markers were calculated using DArTsoft 7.4 (Diversity Arrays Technology P/L, Canberra, Australia). Markers were scored 1 when present in the genomic representation of the sample, 0 when absent, and −9 for missing data when the clustering algorithm deployed in DArTsoft was unable to score the sample with sufficient confidence. For each marker, two quality parameters were computed. The reproducibility parameter was computed by counting the number of mismatches in replicated samples (missing data excluded). The P value, which can vary from 0 to 1, was calculated by dividing the variance of the hybridization intensity between the two clusters (0 versus 1) by the total variance of hybridization intensity of the marker, with high P values denoting reliable markers. Monomorphic markers in the collection were discarded, as were markers with a P value below 0.8 and markers with more than 10% percent missing data. A similarity matrix was then produced using DARwin 5 software [39] to eliminate markers with identical patterns. The Polymorphism Information Content (PIC) was calculated for the remaining markers. The accessions to be genotyped by GBS were chosen using the maximum length subtree procedure available under DARwin5. This method, which is based on allelic combinations rather than on simple allelic richness, prunes the tree of its most redundant units. It therefore minimizes the risk of spurious associations due to the genetic structure of the studied population while limiting possible reductions of allelic diversity [39].

### Genotyping with SNP markers

Genotyping was conducted at Diversity Arrays Technology Pty Ltd. (Australia) using a method of GBS that combines DArT with a next-generation sequencing technique called DArTseq™, previously described by Courtois et al. [35]. The method achieves genome complexity-reduction using PstI/TaqI restriction digests followed by Illumina short-read sequencing. PstI-specific adapters tagged with 96 different barcodes to encode a plate of DNA samples were ligated to the restriction fragments. The resulting products were amplified and checked for quality. The 96 samples were then pooled and run in a single lane on an Illumina Hiseq2000 instrument. The PstI adapters included a sequencing primer so that the tags generated were always read from the PstI sites. The resulting sequences were filtered and split into their respective target datasets, and the barcode sequences were trimmed. The sequences were trimmed at 69 bp (5 bp of the restriction fragment plus 64 bases with a minimum quality score of 10). An analytical pipeline developed by DArT P/L was used to produce DArT score tables and SNP tables. Markers that had no position on the *Nipponbare* sequence and more than 20% missing data were discarded from the initial dataset.

### Population structure

For population structure analyses, we used only the SNP markers. We randomly selected a sub-sample of markers that showed a rate of missing data below 2.5% and a distance to the nearest marker of at least 100 kb. Structure software v2.3.4 developed by Pritchard et al. [40] was used to analyze the organization of the panel. The parameters used were haploid data, burn-in of 200,000 steps, 200,000 iterations, admixture model with correlated frequencies, K varying from 1 to 10 and 10 runs per K value. After discarding the runs that did not converge, the data were analyzed using Structure Harvester [41] which incorporate the criteria developed by Evano et al. [42] that help to determine the number of populations in a panel. To further facilitate this step, the discriminant analysis of principal components (DAPC) method developed by Jombard et al. [43] was also implemented using the R Adegenet package [44]. An accession was discretely assigned to a population when more than 75% of its genomic composition came from that population. The pairwise Wright’s fixation index (F_ST_) values, which measure the genetic differentiation between populations [45], were computed using Arlequin [46] with 1000 permutations to determine their significance. To permit an easy visualization of the relationships between accessions, an unweighted neighbor-joining (NJ) tree was constructed using a dissimilarity matrix. For DArT markers, the matrix was computed using a Sokal and Michener [47] dissimilarity index [dij = u/[m + u]], where u is the number of non-matching alleles between individuals i and j, and m is the number of matching alleles from the DArT matrix. For SNP markers, the matrix was computed using a shared allele index. All analyses were conducted using DARwin software [39]. Population attributions derived from the model-based approach were projected on the graphical tree representation.

In a second and finer-scale round of analysis, the populations detected in the panel were submitted to the same set of analyses using a subset of markers that were polymorphic in the populations studied.

### Linkage disequilibrium

To assess whether the marker density was sufficient for association mapping purposes, the linkage disequilibrium (LD) within the panel was evaluated by computing the r^2^ values between pairs of SNP markers using Tassel v5.0 on a chromosome basis [48]. Because LD is highly affected by panel structure, LD was only computed within each subpanel. LD indices perform poorly with markers with very low allelic frequencies [13]. For this reason, only markers with an MAF above 10% were used. For each marker pair, the physical distance between markers was computed on a chromosome basis. Because of the large variance in the LD estimates of any SNP pair, the marker pairs were discretized in classes of 25 kb physical distance, and the r^2^ values were averaged by class to reduce the effect of outliers, as proposed by Mather et al. [14]. The average r^2^ values were tabulated as a function of the classes of physical distances between markers. A power law (y = ax^k^) was fitted to the data to determine the physical position (x) corresponding to a given r^2^ value (y).

### Plant phenotyping under field conditions

The accessions were grown under field conditions in the Plant Resource Center located at An-Khan-Hoai Duc, near Hanoi (21° 00' 02'' N and 105° 43' 07'' E), Vietnam, during the 2011 wet season. The same plots were used to collect DNA from single plants, to start to measure several key parameters and to harvest seeds for future experiments. The experimental design was a randomized complete block design with 3 replications. The plot size was 1.0 m^2^ with three 1.0-m-long rows and a 0.25-m space between rows and between plants within rows. A 2.5-m broad border composed of plants of the *LT3* variety surrounded the whole experiment. The flowering dates were recorded daily. Based on the time from sowing to flowering, four classes of maturity were established: early (E ≤ 85 d), medium (85 d < M ≤ 105 d), late (105 d < L ≤ 135 d) and very late (VL > 135 d). Seeds were harvested and dried. For each accession, 30 seeds were distributed in a Petri plate, and a high definition image was taken. The image was analyzed using Image J [49], and the lengths and widths of 10 grains were recorded. A length to width ratio was computed. Three classes were established: L/W > 3.0 (A), 2.5 < L/W ≤ 3.0 (B) and L/W ≤ 2.5 (C). The glutinous (G) / non- glutinous (NG) nature of the grains was determined using an iodine test on 10 seeds per accession. The seeds were cut in half and immersed in a solution composed of 0.2% I2 in 2% KI [50]. Development of a dark blue color indicated that the grain was glutinous, whereas a brown color indicated that that it was non-glutinous.

These data were projected onto the NJ trees to assess whether they could help to explain the genetic differentiation within the panels.

### Genome-wide association mapping

To establish a matrix adapted for GWAS, markers with a minor allele frequency (MAF) below 5% were discarded. Missing data were imputed using Beagle v3.3.2 [51]. Beagle applies a Markov model to the hidden states (the haplotype phase and the true genotype) along the chromosome using an EM (Expectation-Maximization) algorithm that iteratively updates model parameters to maximize the model likelihood up to the moment where convergence is achieved.

As an example of the potential of this panel, a GWAS was conducted for the time to flowering successively on the full panel and the two subpanels using Tassel v5.0 [48]. A mixed model was used with control of structure and kinship. The structures of the panel and subpanels were based on the percentages of admixture derived from the Structure analyses (see paragraph on population structure). The respective kinship matrices of the panel and subpanels were computed with Tassel. The threshold to declare an association significant was set at P < 5e-04 for the purpose of comparison between panels.

## Results

### DArT marker-based population structure pattern

Among the 6444 DArT markers that were tested, 619 were polymorphic in our dataset (9.6%). Among these 619 markers, 451 had a reproducibility above 99% and a quality index above 0.80, among which 300 had a call rate above 90%. We tested the markers for their similarity and kept only one copy of the 59 groups of identical markers. The final set was therefore composed of 241 non-redundant markers. The PIC of these markers varied between 5% and 50%, with an average of 40.0%. The distribution of the DArT markers in the genome was reasonably uniform. The number of markers per chromosome was proportional to their relative size in bp (r = 0.78, P = 0.003). Large marker-uncovered zones corresponding to peri-centromeric regions were observed.

**Figure 1:**
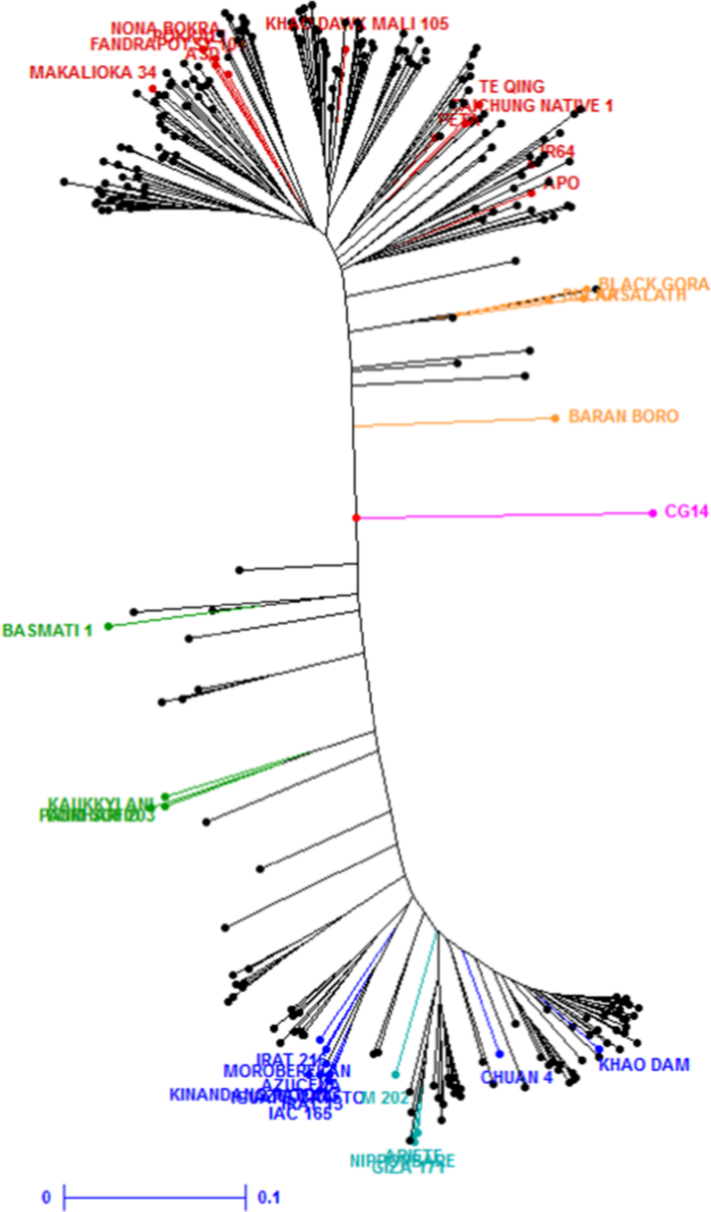
**NJ tree of the 271 accessions based on 241 DArT markers.** The Vietnamese accessions are represented by black dots. In red, *indica* accessions; in yellow, *aus*/*boro* accessions; in green, *sadri*/*basmati* accessions; in dark blue, *tropical japonica* accessions; in light blue, *temperate japonica* accessions. CG14, an *O. glaberrima* accession, in pink, was used as outgroup.

The NJ tree based on the 241 markers clearly showed a bipolar organization (Figure 1). The reference cultivars that were genotyped together with the Vietnamese varieties enabled us to identify the upper part of the graph as *indica* cultivars, the lower part as *japonica* cultivars and the remainder as intermediates, some being close to the *aus*/*bor*o or *sadri*/*basmati* accessions. The Structure analysis confirmed the bipolar organization, with K = 2 as the most likely subgroup number. Among the 270 accessions (*O. glaberrima* excluded), 168 were identified as *indica*, 88 as *japonica*, and 14 as admixed. The match between the tree position and the Structure population attributions was perfect for the *indica* and *japonica* accessions while the *aus*/*bor*o- and *sadri*/*basmati*-like accessions were mostly classified as admixed, with a few *aus*/*boro*-like accessions classified as *indica*. Some accessions clustered at the same position indicating a very high level of similarity. Some of these accessions had similar names (e.g., *Ba Cho Kte* for both G84 and G297), while others were different (e.g., *Ble Blau Da* and *Ble Blau Blau* for G197 and G198).

The DArT data were used to select 182 non-redundant Vietnamese accessions and three reference varieties (*Nipponbare*, a *temperate japonica*; *Azucena*, a *tropical japonica*; and *IR64*, an *indica*). The number of markers derived from this first analysis was clearly insufficient for the purpose of association mapping. We therefore completed the genotyping of the 185 selected accessions using GBS.

### Genotyping-by-sequencing-based population structure pattern

GBS yielded 25,971 markers (15,284 GBS-DArTs and 10,687 SNPs) after the data-cleaning step. The PIC of these markers varied between 1% and 50%, with an average of 32.0%, slightly lower to that of the initial DArTs.

Structure was first run on the whole set of 182 Vietnamese varieties with a subset of 1275 SNP markers. The results confirmed the existence of two groups: 114 *indica* and 62 *japonica* accessions, and 6 admixed accessions (checks excluded). The group attribution was almost identical to that obtained with the DArT markers with a few exceptions: G181 was assigned to the *japonica* subpanel, but here it clustered with the *indica* subpanel. This discrepancy most likely resulted from a mislabeling at some point in the DNA manipulation. One accession initially considered as admixed (G211) was assigned to the *indica* subpanel and, reciprocally, another accession initially considered as *indica* (G207) appeared admixed.

### Characteristics of the indica subpanel

**Figure 2:**
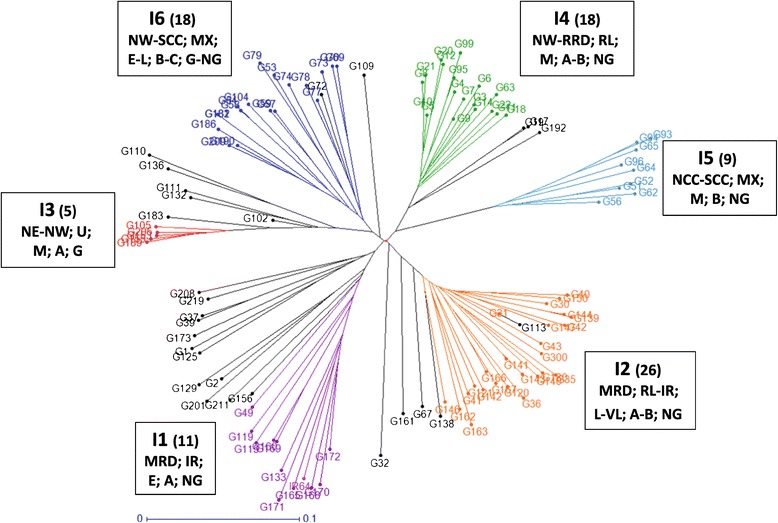
**Populations detected in the** ***indica*** **subpanel.** Accessions belonging to the same population are in the same color; in brackets, the number of accessions of the population; admixed are in black; the *indica* check (*IR64*) is in pink. The main characteristics of the populations, separated by a semi-column, are given in the following order: − Zone of origin: MRD = Mekong River Delta; SE = Southeast; CH = Central Highlands; SCC = South Central Coast; NCC = North Central Coast; RRD = Red River Delta; NW = Northwest; NE = Northeast - Ecosystem: IR = irrigated; RL = rainfed lowland; UP = upland; MX = mixture of types - Maturity class: E = early; M = medium; L = late; VL = very late - Grain length to width ratio: A = L/W > 3.0; B = 2.5 < L/W < =3.0; C = L/W < =2.5 - Grain type: G = glutinous; NG = non-glutinous.

Structure was run on the 114 *indica* accessions with a set of 840 SNP markers. Six populations were detected and confirmed by a DAPC analysis (Additional file 1: Table S1). The populations are represented in Figure 2. The passport information (province and ecosystem) and phenotyping data (maturity time, grain shape and endosperm type) enabled us to characterize these populations (Table 1). Population I1 (11 accessions) included mostly short-duration improved irrigated accessions from the Mekong River delta, all possessing long and slender grains that were generally non-glutinous. Population I2 (26 accessions) included almost exclusively long- and very long-duration rainfed lowland accessions also from the Mekong delta, with a non-glutinous grain type but a large diversity of shapes. Population I3 (5 accessions) was composed of late to very late glutinous upland accessions from the Northeast and Northwest mountain regions, with a rather long and slender grain type. Population I4 (18 accessions) was composed of medium-duration accessions from the Red River delta or the Northwest regions, with rather medium and narrow non-glutinous grains. Population I5 (9 accessions) regrouped medium-duration accessions from various ecosystems of the North and South Central Coast regions, with rather small and non-glutinous grains. Population I6 (18 accessions) was more difficult to characterize. It was composed of a heterogeneous set of accessions from various ecosystems of the Northwest and South Central Coast regions, with a large range of durations, small or medium and mostly non glutinous grain types. The admixed set (27 accessions) did not show any particular characteristics, except a relatively high rate of glutinous accessions.

**Table 1:** **Characteristics of the populations detected by structure**

**Region**	**I1**	**I2**	**I3**	**I4**	**I5**	**I6**	**Im**	**All I**	**J1**	**J2**	**J3**	**J4**	**Jm**	**All J**
Northeast			1	1			5	7	9	2			1	12
Northwest			4	3		5	5	17	19	3				22
Red River Delta		1		10	1		3	17		3		5		8
North Central Coast				1	5	1	2	9	3	1		1	1	6
Central Highlands								0					1	1
South Central Coast	2				3	11	5	20	1	1	5		1	8
Southeast		2					2	4			1			1
Mekong River Delta	7	23					4	34	1					1
na	1			3		1	1	6	3					3
**Ecosystem**	**I1**	**I2**	**I3**	**I4**	**I5**	**I6**	**Im**	**All I**	**J1**	**J2**	**J3**	**J4**	**Jm**	**All J**
Irrigated	8	7	1	5	3		6	30	1	4				4
Mangrove						1		1				4		4
Rainfed lowland	1	12		2	2	9	8	34	3	1		2	1	7
Upland	1	2	4		4	5	9	25	25	1	5		1	30
na	1	5		11		3	4	24	7	4	1		2	17
**Cycle**	**I1**	**I2**	**I3**	**I4**	**I5**	**I6**	**Im**	**All I**	**J1**	**J2**	**J3**	**J4**	**Jm**	**All J**
Very early	3			1			1	5				1		1
Early	5				1	5	6	17	16	2		5	1	24
Medium	2		5	15	8	7	8	45	154	5	2		1	23
Long		6		1		6	10	23	1	2	3		2	8
Very long		20					1	21	1					1
na	1			1			1	3	3	1		1		5
**Grain length (L)**	**I1**	**I2**	**I3**	**I4**	**I5**	**I6**	**Im**	**All I**	**J1**	**J2**	**J3**	**J4**	**Jm**	**All J**
Short		4		1	4	4	3	16		8		3		11
Medium	1	10		14	4	11	12	52	4	2	1	3	1	11
Long	4	8	2	3	1	3	7	28	22		3		3	28
Very long	6	3	3				5	17	9		2			11
na		1						1	1					1
**Grain width (W)**	**I1**	**I2**	**I3**	**I4**	**I5**	**I6**	**Im**	**All I**	**J1**	**J2**	**J3**	**J4**	**Jm**	**All J**
Large					1	1	2	4	25	7	5	1	3	41
Medium	5	13	3	5	4	17	17	64	10	3	1	5		19
Narrow	6	12	2	13	4		8	45					1	1
na		1					1	2	1					1
**L/W ratio**	**I1**	**I2**	**I3**	**I4**	**I5**	**I6**	**Im**	**All I**	**J1**	**J2**	**J3**	**J4**	**Jm**	**All J**
A (>3.0)	10	12	4	6	1		10	43	6		2		1	9
B (2.5 < L/W < =3.0)		10	1	12	7	10	11	51	16		1	2		19
C (<=2.5)	1	3			1	8	6	19	13	10	3	4	3	33
na		1						1	1					1
**Grain type**	**I1**	**I2**	**I3**	**I4**	**I5**	**I6**	**Im**	**All I**	**J1**	**J2**	**J3**	**J4**	**Jm**	**All J**
Glutinous	2	3	5	1	1	4	8	24	22	10		3	3	38
Non glutinous	9	23		17	8	14	19	91	14		6	3	1	24
**Total**	11	26	5	18	9	18	27	114	36	10	6	6	4	62

I = *indica*; J = *japonica*; na = no available data.

### Characteristics of the japonica subpanel

**Figure 3:**
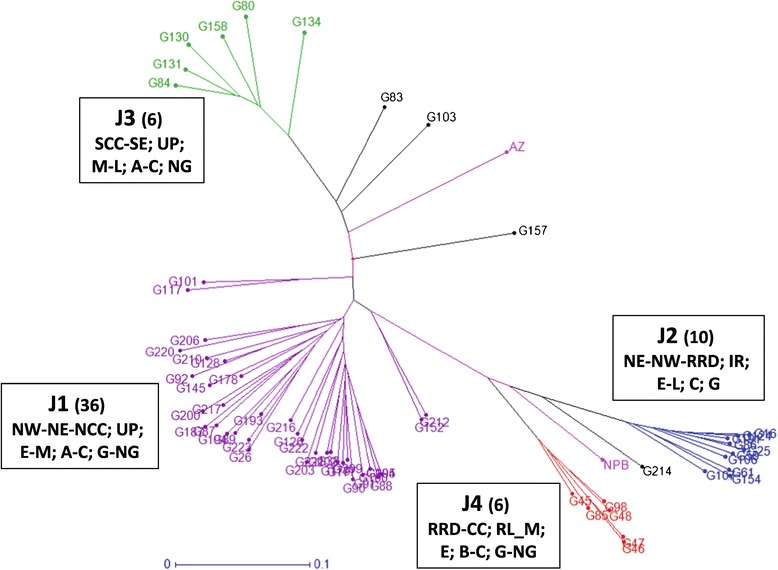
**Populations detected in the** ***japonica*** **subpanel.** Accessions belonging to the same population are in the same color; in brackets, the number of accessions of the subpopulation; admixed are in black; the two japonica checks (*Azucena* and *Nipponbare*) are in pink. The main characteristics of the populations, separated by a semi-column, are given in the following order: − Zone of origin: SCC = South Central Coast; NCC = North Central Coast; NW = Northwest; NE = Northeast- Ecosystem: IR = irrigated; RL = rainfed lowland; UP = upland; M = mangrove - Maturity class: E = early; M = medium; L = late; VL = very late - Grain length to width ratio: A = L/W > 3.0; B = 2.5 < L/W < =3.0; C = L/W < =2.5 - Grain type: G = glutinous; NG = non-glutinous.

The same analysis was performed on the 62 japonica accessions with a set of 780 SNP markers. Four populations and a small admixed set (4 accessions) were detected and are represented in Figure 3. These populations were subsequently characterized using the available passport and phenotypic data (Table 1). Population J1 (36 accessions) was mostly composed of early- and medium-duration upland accessions from the Northeast and Northwest mountainous regions, with a high proportion of glutinous types and the long, large grains typical of the upland varieties from Southeast Asia. Population J2 (10 accessions) was heterogeneous for ecosystems and regions of origin but homogenous for duration (medium) and grain characteristics: all accessions were glutinous with short, large grains (C length to width ratio). Population J3 (6 accessions) regrouped the medium to late accessions from the South Central Coast and Southeast regions, with long, large non-glutinous grains. Population J4 (6 accessions) was composed of early rainfed lowland and mangrove accessions, mostly from the Red River delta, with short or medium grains.

The genetic differentiation among *indica* and *japonica* populations, as measured by F_ST_, was always highly significant (Table 2) but it was higher among *japonica* populations (F_ST_ varying from 0.428 to 0.692) than *indica* populations (0.264 to 0.555). These values are consistent with the group distances shown on Figure 2 (*indica* accessions) and Figure 3 (*japonica* accessions).

**Table 2:** **F**_**ST**_ **among populations within the** ***indica*** **and the** ***japonica*** **subpanels**

***indica***	**I1**	**I2**	**I3**	**I4**	**I5**	**I6**
I1		0.001	0.003	0.001	0.001	0.001
I2	0.303		0.001	0.001	0.001	0.001
I3	0.406	0.453		0.001	0.001	0.001
I4	0.327	0.301	0.498		0.001	0.001
I5	0.374	0.405	0.555	0.381		0.001
I6	0.264	0.270	0.375	0.269	0.347	
***japonica***	**J1**	**J2**	**J3**	**J4**		
J1		0.001	0.003	0.001		
J2	0.528		0.001	0.001		
J3	0.428	0.692		0.001		
J4	0.461	0.542	0.676			

F_ST_ values below the diagonal, probability based on 1000 permutations above the diagonal.

### Linkage disequilibrium

The decay of LD along physical distances was computed for both the indica (114 accessions) and japonica (62 accessions) subpanels. In the indica subpanel, r^2^ was at its maximum (0.52) in the 0–25 kb marker distance interval. R^2^ reached values of 0.2 and 0.1 at 101 kb and 343 kb, respectively (Table 3). The decay was relatively similar for all chromosomes except chromosome 11, for which the decay was faster (Additional file 2: Figure S1). By comparison, LD started at higher values in the 0–25 kb interval in the japonica subpanel (0.71). The LD decay was also much slower with r^2^ reaching 0.2 and 0.1 at 425 kb and 1,783 kb, respectively, and more heterogeneous across chromosomes. As for the indica subpanel, the decay was faster for chromosome 2, but LD hardly decreased below 0.2 for chromosomes 3, 6 and 8 (Additional file 2: Figure S2). These figures describe a general trend that is useful for determining whether the average marker density is sufficient for association mapping purposes. However, in both subpanels, the overall data also showed huge variations in r^2^ for the interval classes with short marker distances. For example, for the 0–25 kb interval, between 11% (japonica subpanel) and 22% (indica subpanel) of the r^2^ values were below 0.10, i.e., a surprisingly high proportion, while 60% and 50% of the r^2^ values were above 0.8, respectively. The low r^2^ values in the 0–25 kb interval were generally attributable to the presence of 0 in the contingency tables, due to a combination of the smaller size of the subpanels and the frequent occurrence of relatively rare alleles. For the intervals above a 1-Mb distance between markers, however, the reverse was not true and high LD values were rare to very rare. These variations in r^2^ indicated that LD around a marker of interest must to be considered at the local level to select candidate genes.

**Table 3:** **Extent of linkage disequilibrium (in kb) in the** ***indica*** **and** ***japonica*** **subpanels**

**Chr**	***indica***		***japonica***	
	**r** ^**2**^ **= 0.1**	**r** ^**2**^ **= 0.2**	**r** ^**2**^ **= 0.1**	**r** ^**2**^ **= 0.2**
1	321	83	2125	180
2	198	60	1614	358
3	370	81	1890	747
4	324	94	1961	261
5	788	306	1065	464
6	378	114	1955	677
7	349	101	1949	452
8	315	70	3314	614
9	264	88	1931	362
10	285	68	1297	390
11	145	35	953	217
12	381	107	1340	375
Average	343	101	1783	425

A power-law (y = ax^k^) was fitted to the data to determine the physical position (x) corresponding to a given r^2^ value (y).

### Genome-wide association mapping result for flowering time

**Figure 4:**
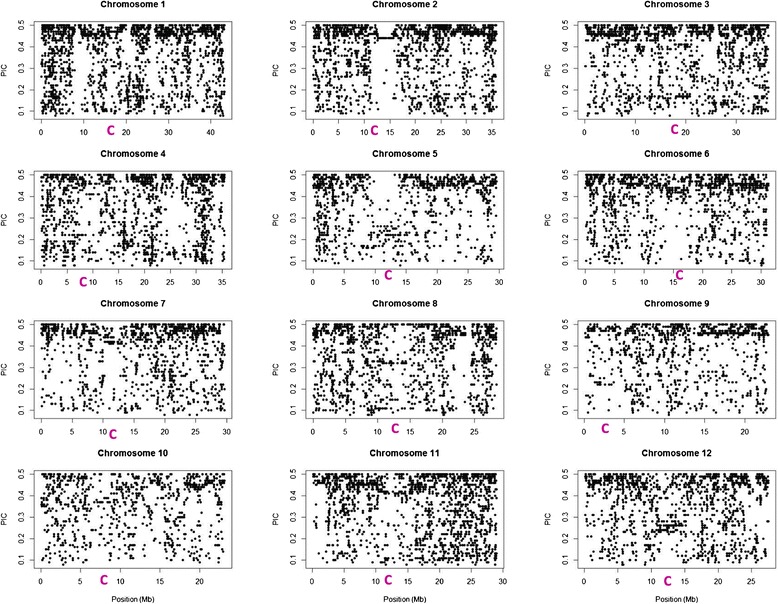
**Distribution of the GBS markers on the 12 chromosomes (distances in Mb) in function of their PIC in the GWAS matrix.** C (in pink): position of the centromere.

For GWAS purposes, the markers with low allele frequency (<5%) were removed from the full panel matrix and the missing data were imputed. The final matrix contained 21,814 markers (12,884 GBS-DArTs and 8,930 SNPs). The markers were distributed in the genome at an average rate of one marker per 17.1 kb (Figure 4). We observed two gaps devoid of markers larger than 500 kb (on chromosomes 1, 6, 7, 8 and 11) and 12 additional smaller gaps of 300 kb to 500 kb (on chromosomes 1, 2 4, 5, 7, 8, and 9). Two other matrices were constituted, one for the indica accessions and one for the japonica accessions, eliminating the markers that became monomorphic or those whose MAF fell below 5% within each panel. These final matrices were composed of 13,979 markers × 114 accessions for the indica subpanel and 8,871 markers × 62 accessions for the japonica subpanel.

The chosen mixed model, which involved both population structure and kinship to account for the effect of population stratification and relatedness, enabled to control the number of false positives in the panel and subpanels as shown by the limited deviations of the cumulative distributions of the observed -log(P-values) to the expected ones on the quantile-quantile plots (Additional file 3: Figure S3). The results of the GWAS are given in Table 4 for the full panel and the two subpanels. The number of significant associations appeared to be linked to the panel size: the larger the panel, the higher the number of associations with greater significance detected. Nineteen, three and two markers appeared significant (P < 5e-04) for the full panel and the indica and japonica subpanels, respectively. One marker specific for the indica panel was detected, while the two markers that were significant in the japonica subpanel were also detected in the full panel.

**Table 4:** **Significant associations detected for flowering time in the full panel and the two subpanels**

**Panel**	**Marker**	**Chr**	**Position**		**P**
Full	D01_15848291R	1	15 848 291		5.51E-05
Full	D01_20832995R	1	20 832 995		1.80E-04
Full	**S01_32217904R**	**1**	**32 217 904**		2.39E-06
Full	D01_39282424R	1	39 282 424	39 422 116	3.72E-04
Full	S02_08797473R	2	8 797 473		2.01E-04
Full	S02_20869087F	2	20 869 087		3.15E-04
Full	D02_22452791R	2	22 452 791		7.19E-05
Full	D03_34777973R	3	34 777 973		1.93E-05
Full	**D04_29486930F**	**4**	**29 486 930**		4.51E-04
Full	D04_32049072F	4	32 049 072		2.84E-04
Full	D05_01172704F	5	1 172 704		3.29E-07
Full	S06_07699940F	6	7 699 940		2.32E-04
Full	**S06_08107519R**	**6**	**8 107 519**		2.31E-04
Full	S06_24762717R	6	24 762 717		9.79E-06
Full	**S06_25132524F**	**6**	**25 132 524**		1.68E-04
Full	S07_21215197R	7	21 215 197		3.03E-04
Full	D09_21763749F	9	21 763 749	21 763 752	3.43E-04
Full	S10_14744127F	10	14 744 127	14 746 792	6.11E-05
Full	D11_20936368R	11	20 936 368		2.17E-06
*Indica*	D01_04765339R	1	4 765 339		2.35E-04
*Indica*	**D04_29486930F**	**4**	**29 486 930**	29 543 971	6.65E-05
*Indica*	**D06_07966086R**	**6**	7 966 086	**8 107 519**	3.70E-04
*Japonica*	**S01_32217904R**	**1**	**32 217 904**		4.37E-04
*Japonica*	**S06_25132524F**	**6**	**25 132 524**		8.53E-05

In bold associations significant in more than one panel.

## Discussion

For the purpose of conducting GWAS in the future, we developed a panel of 182 traditional Vietnamese accessions and performed a high density genotyping with DArT and SNP markers.

We first created a library of 6444 DArT clones from a mixture of indica and japonica cultivars. The method to genotype DArT markers was said to be technologically challenging [25], but in our case, it gave reasonable results with high reproducibility. The method used to identify the polymorphic markers tends to select against markers with low MAF because they are, by construction, associated with lower P values, one of the two quality indices for DArT. This selection explains the high PIC average (40%). If the rare occurrence of low MAF markers can complicate the discrimination of minority groups in diversity studies [28], it is advantageous for GWAS in that those markers are in any case eliminated because the power of markers with low MAF to detect associations is limited [52]. The challenge appears to mainly lie in the throughput of the method; although the potential number of clones in the library (6,444) is high enough for GWAS purposes, the number of useful clones turned out to be much lower. The percentage of polymorphisms, approximately 10%, was very low for a mixture of indica and japonica accessions, although the figure is close to what was observed in a similar background (i.e., 14.5% by Jaccoud et al. [24]). The number of usable marker also decreased for other reasons, such as quality and marker redundancy. The number of remaining markers was sufficient to conduct a first analysis of the genetic diversity of the panel and to select non-redundant accessions. However, it was insufficient for GWAS.

In contrast, the final number of GBS markers obtained (21,623) is well adapted to GWAS purposes. The technical simplicity and reduced cost of this method account for the increasing interest it has attracted [32]. The main difficulties lie in the extraction of the marker × accession matrix from the raw sequence reads, which implies the use of a bioinformatics pipe-line such as the one developed by Glaubiz et al. [53], which requires reasonable expertise in its parametrization, notably for proper heterozygote identification. Fortunately, our material was composed of highly homozygous lines, and this difficulty did not apply. The rate of missing data can be important with GBS [32]. To reduce genome complexity, we used PstI, which is a rare-cutter restriction enzyme (6 bp recognition site) that results in a smaller library and better depth of coverage of the library in the sequence data than frequent cutters such as ApeKI. Even with the choice of a rare-cutter enzyme, the threshold of less than 20% missing data that we applied to determine whether to keep a marker in the dataset led us to remove a large number of markers and implied imputation for the remaining scattered missing data. Imputation is considered an efficient and cost-effective way to deal with missing data – the alternative being sequencing at a higher depth – but, to be truly accurate, it supposes that the LD spans a long distance, the marker order is correct, the marker density is sufficiently high and that all haplotypes that need to be imputed are captured in the initial dataset [54]. All these conditions appear to be reasonably fulfilled in our panel. Even if the marker distribution, determined by the repartition of the restriction enzyme cut sites, still left a few missing segments in the genome, as a whole, the panel is covered by a large number of well distributed markers.

The genetic diversity of cultivated rice long ago became an object of detailed studies with molecular markers [55]. However, because the sampling was worldwide in these studies, few Vietnamese varieties were included in the studied panels. The analysis of a much larger sample of accessions from Vietnam permitted a finer segmentation of the accessions into populations, as also achieved by Myint et al. [56] and Radanielina et al. [57], who both identified new specific groups in accessions from Myanmar and Madagascar, respectively. Available passport information (province and ecosystem) from the genebank and phenotyping data (maturity time, endosperm type and grain shape) acquired in the framework of this study enabled us to further characterize some of the populations. This combination of traits is commonly used to classified rice germplasm in Asia [58-60].

The global genetic structure of our panel of Vietnamese varieties was nearly reduced to the *indica*-*japonica* binarity, with very few accessions that were classified as intermediate between the *indica* and *japonica* sub-species. This pattern is consistent with current knowledge regarding the global genetic organization of *O. sativa* in this region [3,19]. At a finer scale of analysis, the *japonica* subpanel appears more highly structured than the *indica* subpanel. This can be seen in the shape of the diversity trees (star-like for the *indica* subpanel and long branches for the japonica *subpanel*), among population F_ST_ values (higher on average for the *japonica* populations), and the proportion of admixed accessions (lower for the *japonica* subpanel). This stronger structuration is partly due to the co-existence in the *japonica* subpanel of a *tropical* and a *temperate* compartment to which groups J1 and J2, respectively belong. Population J1, which clusters with *Khao Dam* in the global diversity tree, has all of the characteristics of *tropical japonica* varieties from Southeast Asia (upland ecosystem, origin from mountainous provinces, long and large grains). The features of population J2 (irrigated ecosystem, origin in mountainous provinces, short and round grains) as well as the fact that it clusters with *Nipponbare*, *Ariete* and *Giza 171* support the conclusion that this population is part of the *temperate* compartment. In the tropics, *temperate japonica* varieties can be found in cold-prone situations at high elevations, but mostly under irrigated systems because water is needed to buffer temperature variations. Fukuoka et al. [3] also identified, within a set of accessions from North Vietnam, a group that they classified as *temperate japonica*. Populations J3 and J4, although relatively homogeneous genetically, are small in size and heterogeneous for the phenotypic data and therefore more difficult to qualify with the available elements.

Within the *indica* populations, I1 can easily be equated to modern high-yielding varieties and I2 to rainfed lowland photoperiod-sensitive varieties grown in medium-deep flood-prone areas of the South delta, following Bong's classification [61]. I3 constitutes a small atypical group (upland ecosystem, long and narrow glutinous grains) with the highest F_ST_ with the other groups. For the remaining groups, the characterization is much less obvious, as exemplified by population I6, which is heterogeneous for all traits.

Although passport data and the phenotyping undertaken herein helped to characterize the populations to some extent, information that could help to understand the genetic differentiation is still lacking. For instance, knowing the precise elevation of the accession collection site would be useful because the average altitude of an administrative district may not represent it well. The target cropping season is generally unknown while Ishii et al. [60] differentiated between "fifth month rices" grown during the dry season and "tenth month rices" grown during the rainy season in North Vietnam. The absence of this information does not permit us to associate our populations with these subgroups.

One finding that appears somewhat surprising is the number of *indica* accessions used in the upland ecosystem, such as those from population I3. *Aus* and *indica* accessions are dominant in the upland areas of South Asia and equatorial Asia, but, in Southeast Asia, *tropical japonica* types are generally grown in the uplands [6,19,59,62]. The accuracy of ecosystem attribution can thus be questioned, reinforced by the fact that this information is absent for a large proportion of accessions. Passport data should be regarded with caution, notably when the data are based on off-season collecting missions involving the acquisition of samples from threshing floors or farmers' grain stores [58]. However, previous isozyme data obtained for a collection of upland rice varieties from all over Vietnam also showed that approximately one fourth of the upland rice varieties were *indica*, possibly because of the existence of conditions more favorable to upland rice than in neighboring countries or because of higher exchanges with zones of irrigated rice [5].

Southeast Asia is characterized by a frequent preference by certain ethnic groups for glutinous rice as a staple, not just as a dessert. In Laos, for example, glutinous rice accounts for 85% of the production [63]. While the situation is not as extreme in Vietnam, glutinous grain types still represent 33% of the accessions in the full panel, with a frequency of glutinous endosperms higher within the *japonica* subpanel (61% of the accessions) than within the indica *subpanel* (21%). Some populations, such as I3 and J2, are exclusively composed of glutinous types. The intron 1 splice site mutation in the *Waxy* gene, which is responsible for the change in endosperm type, has a single evolutionary origin and most likely arose within the *tropical japonic*a before being introgressed in the *indica*, explaining why both groups share the same mutation [64].

Strong differences in genomewide LD decay were observed between the *indica* and *japonica* subpanels. These differences were also encountered in other collections [14] and are due to differences in evolutionary history between the two sub-species, notably in the severity of the domestication bottleneck and further demographic and hybridization events. The *japonica* sub-species is said to have been domesticated in South China, while opinions differ on the origin of the *indica* subspecies [65-67]. The slower LD decay observed in the *japonica* subpanel may be attributable to the stronger structure observed within this panel, with the presence of four well-differentiated populations. Within a given panel, variations in LD decay between chromosomes can be linked to the presence on specific chromosomes of genes that underwent selective sweeps [68] or differences in recombination rates affected by the proportion of repetitive DNA or structural differences. In any case, the final average marker density (one marker per 17.1 kb) is higher than the r^2^ decay and therefore suitable for GWAS It is in fact safer to reach an average marker density higher than that required by the LD decay range because LD decay estimates have a large variance, markers are not absolutely evenly distributed all over the genome, and some markers have very low MAF and are therefore less powerful at detecting associations.

The results of the GWAS for flowering time illustrate what can be expected from the panel and subpanels. When the phenotypic variability is not fully linked to the structure, it appears wiser to use the full panel, which leads to a higher number of associations with higher significance. The subpanels, because of their much smaller size, particularly for the *japonica* subpanel, show less detection power, although a significant marker specific for the *indica* subpanel was detected. Compared with the position of the known genes controlling flowering in rice (approximately 40), only marker S10_14744127F on chromosome 10, significant in the full panel, colocalizes with one of these genes. The position corresponds almost perfectly to that of the gene RID1, also named Ehd2 (Os10g28330), located between positions 14 739 569 and 14 739 569, which encodes a master switch from vegetative to floral development in rice [69]. The reason why this specific marker was not detected in either of the two subpanels is because this specific marker has separate *japonica* and *indica* alleles. Therefore, the marker is not polymorphic in the subpanels.

## Conclusions

A panel characterized with a marker density adapted for GWAS is now available for public use [seeds from PRC's co-author and GBS data from TropGeneDB (see section on “Availability of supporting data section” for access)] and constitute a highly valuable resource for mining new alleles within Vietnamese genetic resources.

### Availability of supporting data

The GBS dataset (hapmap format) supporting the results of this article has been deposited as a downloadable Excel file in TropGeneDB: http://tropgenedb.cirad.fr/tropgene/JSP/interface.jsp?module=RICE tab “studies”, study type “genotype”, study “Vietnamese panel - GBS data”.

## Additional files

Additional file 1: Table S1. List of the Vietnamese accessions, with their province of origin, varietal type, population assignments based on DArT and GBS markers, and phenotypic characteristics. **Table S2.** List of the reference accessions included in the analyses. **Table S3.** List of the accessions used to build the DArT library. Additional file 2: Figure S1. Decay of r^2^ along physical distance [in kb] in the indica panel. R^2^ was averaged across marker pairs for 25 kb intervals. **Figure S2.** Decay of r^2^ along physical distance [in kb] in the japonica panel. R^2^ was averaged across marker pairs for 25 kb intervals. Additional file 3: Figure S3. Quantile-quantile plots for the full panel (A), the indica panel (B) and the japonica panel (C).
