# Supplementary material for: Characterization of a panel of Vietnamese rice varieties using DArT and SNP markers for association mapping purposes
Source: BMC Plant Biol. 2014 Dec 19;14:371. doi: 10.1186/s12870-014-0371-7 (PMC4279583; doi:10.1186/s12870-014-0371-7)
Supplement: Additional file 1: Table S1. — List of the Vietnamese accessions, with their province of origin, varietal type, population assignments based on DArT and GBS markers, and phenotypic characteristics. Table S2. List of the reference accessions included in the analyses. Table S3. List of the accessions used to build the DArT library. [file 12870_2014_371_MOESM1_ESM.docx]

Table S1: List of the Vietnamese accessions, with their province of origin, varietal type, population assignments based on DArT and GBS markers, and phenotypic characteristics

|  |  | PRC |  |  |  |  | DArT |  | GBS | Mat | Gr | Gr | L/W | Gr |
| --- | --- | --- | --- | --- | --- | --- | --- | --- | --- | --- | --- | --- | --- | --- |
| ID | Name | No | Province | Zone | Type | Eco | P | GBS | P | class | length | width | ratio | type |
| G1 | TEP HAI PHONG | 11 | HAIPHONG | RRD | T | IR | I | y | Im | VE | M | N | B | NG |
| G10 | TAM SON NAM DINH | 216 | NAM DINH | RRD | T | IR | I | y | I4 | M | M | N | B | NG |
| G100 | KHAU QUAI DANG 2 | 6969 | TUYEN QUANG | NE | T | UP | J | y | J1 | E | L | M | A | NG |
| G101 | DIEO KBIN | 7295 | DAK LAK | SCC | T | UP | J | y | J1 | L | L | L | C | G |
| G102 | TZO KOH DANG 2 | 7303 | THUA THIEN-HUE | NCC | T | u | I | y | Im | M | L | M | A | NG |
| G103 | CU PUA DANG 1 | 7304 | THUA THIEN-HUE | NCC | T | u | J | y | Jm | L | L | N | A | NG |
| G104 | CU PUA DANG 2 | 7305 | THUA THIEN-HUE | NCC | T | u | I | y | I6 | M | M | M | B | NG |
| G105 | NEP THAI LAN | 7312 | HA GIANG & TUYEN QUANG | NE | T | IR | I | y | I3 | M | L | N | A | G |
| G106 | NEP HAI HAU | 7316 | NINH BINH | RRD | T | IR | J | y | J2 | L | S | M | C | G |
| G107 | NEP THAI BINH LUN | 7317 | NINH BINH | RRD | T | IR | J | y | J2 | L | S | M | C | G |
| G108 | TAM AP BE | 7318 | NINH BINH | RRD | T | IR | m | y | m | L | M | N | A | NG |
| G109 | MANH GIE | 7349 | QUANG BINH | NCC | T | UP | I | y | Im | L | M | M | B | NG |
| G11 | TAM TRON HAI DUONG | 219 | HAI DUONG | RRD | T | u | I | y | I4 | M | S | N | B | NG |
| G110 | RAN TRANG | 7823 | BINH THUAN | SE | T | UP | I | y | Im | L | M | M | B | NG |
| G111 | NEP RAY | 7824 | BINH THUAN | SE | T | UP | I | y | Im | L | VL | N | A | G |
| G112 | NEP LUA | 7826 | VUNG TAU | SE | T | IR | I | n |  | VL | L | N | A | na |
| G113 | NANG THIET | 7827 | VUNG TAU | SE | T | IR | I | y | I2 | L | M | N | A | NG |
| G114 | NEP QUA | 7830 | KHANH HOA | SCC | T | UP | I | n |  | L | VL | M | A | na |
| G115 | KOI LOI | 7910 | HA NOI | RRD | T | IR | I | y | I1 | E | M | M | C | NG |
| G116 | KOI PU | 7913 | THUA THIEN HUE | NCC | T | u | I | n |  | L | VL | M | A | NG |
| G117 | KHAO SANG | 7930 | QUANG TRI | NCC | T | UP | J | y | J1 | M | VL | L | B | G |
| G118 | L03 | 9175 | HA NOI | RRD | I | IR | I | n |  | M | VL | L | C | G |
| G119 | L26 | 9198 | HA NOI | RRD | I | IR | I | y | I1 | na | VL | M | A | G |
| G12 | TAM CAO VINH PHUC | 226 | VINH PHUC | RRD | T | IR | I | y | I4 | M | M | N | A | NG |
| G120 | BAY THANH | 9219 | CA MAU | MRD | T | IR | I | y | I2 | VL | L | N | A | NG |
| G121 | CA RO | 9246 | TAY NINH | SE | T | IR | I | y | I2 | VL | M | M | B | NG |
| G122 | NEP LAO TRANG | 9252 | DAC LAC | SSC | T | UP | I | n |  | na | L | L | C | na |
| G123 | BAO THAI LUN | 9353 | QUANG NINH | NE | T | IR | I | n |  | na | S | M | C | na |
| G124 | NEP DEN | 9355 | QUANG NINH | NE | T | IR | J | y | J2 | M | S | L | C | G |
| G125 | NEP NUONG | 9356 | QUANG NINH | NE | T | u | I | y | Im | E | M | M | C | G |
| G126 | KHAU DAM DOI | 9466 | NGHE AN | NCC | T | UP | J | y | J1 | M | VL | L | B | G |
| G127 | KHAU BAO THAI | 9470 | NGHE AN | NCC | T | IR | I | n |  | na | S | M | C | na |
| G128 | KHAU DAM | 9476 | NGHE AN | NCC | T | UP | J | y | J1 | M | L | L | C | G |
| G129 | LC93-2 | 9507 | KHANH HOA | SCC | T | UP | I | y | Im | E | L | M | B | NG |
| G13 | TAM DEN HAI PHONG | 253 | HAIPHONG | RRD | T | IR | I | n |  | M | S | N | B | NG |
| G130 | LUA DA BO | 9509 | KHANH HOA | SCC | T | UP | J | y | J3 | L | L | L | C | NG |
| G131 | PADAI LONG KHANH | 9517 | KHANH HOA | SCC | T | UP | J | y | J3 | L | VL | L | A | NG |
| G132 | PADAI TLIG JUG | 9520 | KHANH HOA | SCC | T | UP | I | y | Im | L | VL | M | A | G |
| G133 | A 330 | 9524 | KHANH HOA | SCC | I | IR | I | y | I1 | E | VL | M | A | NG |
| G134 | PADAI CALOC | 9530 | KHANH HOA | SCC | T | UP | J | y | J3 | na | M | L | C | NG |
| G135 | PADAI DLUC | 9532 | KHANH HOA | SCC | T | UP | J | n |  | VE | L | L | C | NG |
| G136 | PHUOC LONG | 9541 | KHANH HOA | SCC | T | UP | I | y | Im | L | M | M | B | NG |
| G137 | NEP NGHE | 9554 | BEN TRE | MRD | T | RL | I | n |  | VL | L | L | C | G |
| G138 | NANG QUAT | 9563 | BEN TRE | MRD | T | RL | I | y | Im | L | L | N | A | NG |
| G139 | LUA NANG DEN | 9568 | BEN TRE | MRD | T | RL | I | y | I2 | VL | M | M | C | NG |
| G14 | TAM NHO BAC NINH | 318 | BAC NINH | RRD | T | IR | I | y | I4 | M | M | N | A | NG |
| G140 | LUA BAY DANH | 9570 | BEN TRE | MRD | T | RL | I | y | I2 | L | L | M | B | NG |
| G141 | LUA NANG NIEU CHUM | 9573 | BEN TRE | MRD | T | RL | I | y | I2 | VL | M | M | B | NG |
| G142 | LUA THANH TRA | 9574 | BEN TRE | MRD | T | RL | I | y | I2 | VL | VL | N | A | NG |
| G143 | NEP TROI CHO | 9576 | BEN TRE | MRD | T | RL | I | y | I2 | L | L | M | A | NG |
| G144 | LUA MUA DIA PHUONG | 9578 | BEN TRE | MRD | T | RL | I | y | I2 | VL | S | M | B | NG |
| G145 | BA RIA | 9580 | BEN TRE | MRD | T | RL | J | y | J1 | VL | M | M | B | NG |
| G146 | NANG LOAN HAT TRON | 9584 | BEN TRE | MRD | T | RL | I | y | I2 | L | L | M | B | NG |
| G147 | LUA NANG LOAN HAT DAI | 9585 | BEN TRE | MRD | T | IR | I | y | I2 | VL | VL | N | A | NG |
| G148 | TRANG TEP | 9588 | BEN TRE | MRD | T | RL | I | n |  | VL | L | M | B | NG |
| G149 | MOT TEP AN GIANG | 9593 | BEN TRE | MRD | T | RL | I | n |  | VL | M | N | A | na |
| G150 | NEP DIA PHUONG | 9595 | BEN TRE | MRD | T | IR | I | y | I2 | L | M | M | B | G |
| G152 | LOC SOM | 9871 | BAC GIANG | NE | T | IR | J | y | J1 | E | M | L | C | G |
| G153 | TE NUONG | 9874 | SON LA | NW | T | UP | I | y | I3 | M | VL | M | A | G |
| G154 | NEP THOM | 9878 | HA TAY | RRD | T | IR | J | y | J2 | E | S | L | C | G |
| G155 | KHAU PE LANH | 9908 | SON LA | NW | T | UP | I | y | I3 | M | VL | M | B | G |
| G156 | LUA K | 9967 | HA NOI | u | T | IR | I | y | Im | M | L | M | B | NG |
| G157 | SO CRIOONG | 9984 | SEKONG | CH | T | u | J | y | Jm | M | L | L | C | G |
| G158 | VA TAI ANA ACU | 12049 | NINH THUAN | SE | T | UP | J | y | J3 | M | L | L | C | NG |
| G16 | NEP VAN RUONG HOA BINH | 384 | HOA BINH | NW | T | u | J | y | J2 | M | M | M | C | G |
| G160 | JASMINE | 12059 | AN GIANG | MRD | I | UP | I | y | I1 | M | VL | M | A | NG |
| G161 | BN1 | 12066 | AN GIANG | MRD | I | IR | I | y | Im | L | VL | N | A | NG |
| G162 | NEANG CON | 12068 | AN GIANG | MRD | T | UP | I | y | I2 | VL | S | N | B | NG |
| G163 | CA CHOCH CHAP | 12071 | AN GIANG | MRD | T | UP | I | y | I2 | VL | VL | N | A | G |
| G165 | GIONG 90 NGAY | 12083 | KIEN GIANG | MRD | T | RL | I | y | I1 | E | VL | N | A | NG |
| G166 | CHIN TEO | 12086 | KIEN GIANG | MRD | T | IR | I | y | I2 | VL | L | N | A | NG |
| G167 | THAN NONG MUA | 12088 | KIEN GIANG | MRD | T | RL | I | y | I2 | L | L | N | A | NG |
| G168 | OM1490 | 12101 | KIEN GIANG | MRD | I | IR | I | y | I1 | VE | L | N | A | NG |
| G169 | JASMINE 95 | 12102 | KIEN GIANG | MRD | I | IR | I | y | I1 | E | VL | N | A | NG |
| G17 | NEP GA GAY HAI DUONG | 394 | HAI DUONG | RRD | T | IR | I | y | Im | M | M | N | A | NG |
| G170 | OM 504 JAPAN | 12103 | KIEN GIANG | MRD | I | IR | I | y | I1 | VE | L | N | A | NG |
| G171 | NEP THAI | 12104 | KIEN GIANG | MRD | T | IR | I | y | I1 | E | VL | N | A | G |
| G172 | VND 95-20 | 12105 | KIEN GIANG | MRD | I | IR | I | y | I1 | VE | L | N | A | NG |
| G173 | TAM THOM TRUNG QUOC | 12107 | KIEN GIANG | MRD | T | IR | I | y | Im | E | M | N | A | NG |
| G177 | CHAM HOM | 12563 | HOA BINH | NW | T | u | J | y | J1 | M | L | M | A | NG |
| G178 | KHAU CHINH PHU | 12573 | HOA BINH | NW | T | u | J | y | J1 | M | L | L | C | G |
| G179 | BLAO PU LAU | 12581 | HOA BINH | NW | T | u | J | y | J1 | M | L | M | A | NG |
| G18 | NEP QUYT HAI DUONG | 407 | HAI DUONG | RRD | T | IR | I | y | I4 | M | M | N | A | G |
| G180 | CA DUNG HAT | 12637 | HA NOI | RRD | T | IR | I | y | I2 | VL | M | M | B | NG |
| G181 | BLAU PLAN PIENG | 12970 | SON LA | NW | T | UP | J | y | I6 | M | M | L | C | G |
| G182 | KHAU MO | 13008 | SON LA | NW | T | UP | I | y | I6 | E | S | M | B | NG |
| G183 | KHAU PE LANH | 13076 | SON LA | NW | T | UP | I | y | Im | E | L | M | A | G |
| G185 | KHAU PE LANH | u | SON LA | NW | T | UP | I | n |  | E | L | M | B | na |
| G186 | KHAU NO | 13309 | SON LA | NW | T | UP | I | y | I6 | E | L | M | B | NG |
| G187 | KHAU DUONG PHUONG | 13320 | SON LA | NW | T | UP | J | y | J1 | M | L | L | C | G |
| G189 | KHAU NAM RINH | 13362 | DIEN BIEN | NW | T | UP | I | y | I3 | M | L | M | A | G |
| G19 | ON | 509 | HA NOI | RRD | T | IR | I | y | Im | M | M | M | B | NG |
| G190 | PLE PHMA CHUA | 13363 | DIEN BIEN | NW | T | UP | I | y | I6 | E | M | M | B | NG |
| G191 | KHAU TAN | 13422 | DIEN BIEN | NW | T | RL | J | y | J2 | na | S | L | C | G |
| G192 | KHAU BAO THAI | 13423 | DIEN BIEN | NW | T | RL | I | y | Im | E | S | M | C | NG |
| G193 | BLE PE XA | 13424 | DIEN BIEN | NW | T | UP | J | y | J1 | E | VL | L | C | G |
| G194 | BLE BLAU LIA | 13425 | DIEN BIEN | NW | T | UP | J | y | J1 | E | VL | L | B | G |
| G195 | BLE BDE | 13426 | DIEN BIEN | NW | T | UP | J | y | J1 | E | L | M | A | NG |
| G196 | BLE CO PON | 13427 | DIEN BIEN | NW | T | UP | m | y | m | E | L | M | B | NG |
| G197 | BLE BLAU BLAU | u | DIEN BIEN | NW | T | UP | J | n |  | M | L | L | B | na |
| G198 | BLE BLAU DA | 13429 | DIEN BIEN | NW | T | UP | J | n |  | E | VL | L | B | G |
| G2 | TA CO LAO CAI | 58 | LAO CAI | NE | T | u | I | y | Im | M | M | M | B | NG |
| G20 | TE LE HOA BINH | 553 | HOA BINH | NW | T | u | I | y | I4 | M | M | N | B | NG |
| G200 | CHA FU NU | 13431 | LAI CHAU | NW | T | UP | J | y | J1 | E | L | L | C | G |
| G201 | CHA XU PHU LU | 13435 | LAI CHAU | NW | T | UP | I | y | Im | M | M | N | A | NG |
| G202 | NONG TO | 13442 | LAI CHAU | NW | T | UP | J | y | J1 | M | L | M | B | NG |
| G203 | PLAU CA BANH | 14212 | DIEN BIEN | NW | T | UP | J | y | J1 | E | L | M | A | NG |
| G204 | PLE DO | 14215 | DIEN BIEN | NW | T | UP | J | y | J1 | E | L | M | A | NG |
| G205 | BLE BLAU CHO | 14251 | SON LA | NW | T | UP | I | y | I3 | M | VL | N | A | G |
| G206 | BLE BLAU DO | 14252 | SON LA | NW | T | UP | J | y | J1 | E | VL | L | B | G |
| G207 | KHAU LUA | 14278 | SON LA | NW | T | RL | I | y | m | E | VL | M | A | G |
| G208 | KHAU BOONG LAM | 14279 | SON LA | NW | T | RL | I | y | Im | E | M | L | C | G |
| G209 | BLE CHO | 14386 | LAI CHAU | NW | T | UP | I | y | I6 | E | S | M | C | NG |
| G21 | GIE TRANG HOA BINH | 614 | HOA BINH | NW | T | u | I | y | I4 | M | M | N | A | NG |
| G210 | KHAU LECH | 14408 | LAO CAI | NE | T | UP | J | y | J1 | E | L | L | C | G |
| G211 | PLAU NGOANG PLAC | 14587 | LAO CAI | NE | T | UP | m | y | Im | M | L | M | B | G |
| G212 | PLAU BULAT | 14589 | LAO CAI | NE | T | UP | J | y | J1 | na | M | L | C | NG |
| G213 | PLAU CA CHAT | u | LAO CAI | NE | T | UP | I | n |  | E | S | M | C | NG |
| G214 | BLE BLAU DO | 14596 | LAO CAI | NE | T | RL | J | y | Jm | E | M | L | C | G |
| G215 | BLE BLAU XA | u | LAO CAI | NE | T | UP | J | n |  | E | L | L | B | G |
| G216 | TOM BEO BUA | 14607 | LAO CAI | NE | T | RL | J | y | J1 | E | M | M | B | NG |
| G217 | BLE BLAU SOA | 14615 | LAO CAI | NE | T | RL | J | y | J1 | E | VL | L | B | G |
| G219 | KHAU LA LANH | 14792 | SON LA | NW | T | RL | I | y | Im | na | L | L | B | NG |
| G22 | TRUNG TRANG TUYN QUANG | 760 | TUYEN QUANG | NE | T | u | I | y | I4 | na | M | N | B | NG |
| G220 | PLE LA | T5300 | LAI CHAU | NW | T | UP | J | y | J1 | E | L | M | B | NG |
| G221 | KHAU MAC CO | T5455 | LAI CHAU | NW | T | UP | J | y | J1 | M | VL | L | B | G |
| G222 | PLE MA MU | T6404 | YEN BAI | NE | T | UP | J | y | J1 | na | L | L | B | NG |
| G223 | BLE BLAU TAN | T6794 | LAO CAI | NE | T | UP | J | y | J1 | E | L | L | B | G |
| G24 | TAM XOAN HAI HAU | 1048 | NAM DINH | RRD | T | IR | m | y | m | L | S | N | B | NG |
| G25 | NEP VANG ONG LAC SON HB | 1058 | HOA BINH | NW | T | u | J | y | J2 | M | S | L | C | G |
| G26 | KHAU CAI NOI | 1325 | TAY BAC | NW | T | u | J | y | J1 | M | L | L | C | G |
| G27 | DOAN KET | 1421 | CAO BANG | NE | T | u | I | n |  | M | M | N | B | NG |
| G28 | KHAU PE LANH | u | BAC THAI | NE | T | u | J | n |  | E | L | M | B | na |
| G29 | NEP CON | 1427 | BAC THAI | NE | T | UP | I | n |  | M | M | M | C | G |
| G295 | CTO VIND IET6155 | u | u | u | T | u | I | n |  | VE | na | na | na | NG |
| G297 | BA CHO KTE | 3525 | BINH DINH | SCC | T | UP | J | n |  | na | na | na | na | na |
| G298 | TAN NGAN | 3588 | YEN BAI | NE | T | RL | J | n |  | na | na | na | na | na |
| G299 | BLAO SINH SAI | 4806 | HOA BINH | NW | T | UP | J | y | J1 | na | na | na | na | G |
| G3 | AN TU VO DO | 85 | HA NOI | RRD | T | IR | I | y | I4 | M | M | N | A | NG |
| G30 | TIEU CHET 5 | 1625 | CAN THO | MRD | T | u | I | y | I2 | VL | L | N | A | NG |
| G300 | NANG QUOT BIEN | u | BAC LIEU | MRD | T | u | I | y | I2 | VL | na | na | na | NG |
| G31 | NANG CHI | 1629 | CAN THO | MRD | T | u | I | y | I2 | VL | M | M | B | NG |
| G32 | NANG DUM | 1633 | CAN THO | MRD | T | u | I | y | Im | VL | S | M | C | NG |
| G33 | CHET CUT | u | CAN THO | MRD | T | u | I | n |  | VL | S | N | B | na |
| G34 | NANG DUM | u | CAN THO | MRD | T | u | I | n |  | VL | S | N | B | na |
| G35 | SANAKI | 1638 | CAN THO | MRD | na | u | I | y | I2 | VL | S | M | C | NG |
| G36 | NANG TAY | 1643 | CAN THO | MRD | T | u | I | y | I2 | VL | S | M | C | NG |
| G37 | NEP CAM | 1845 | HA GIANG | NE | T | RL | I | y | Im | L | VL | N | A | G |
| G38 | NEP NUONG | 1849 | HA GIANG | NE | T | UP | J | y | J1 | M | L | L | C | G |
| G39 | NEP CAM | 1851 | HA GIANG | NE | T | RL | I | y | Im | L | VL | M | B | G |
| G4 | NHONG DO HAI DUONG | 135 | HAI DUONG | RRD | T | u | I | y | I4 | M | M | M | B | NG |
| G40 | NEP DO | 2307 | KIEN GIANG | MRD | T | RL | I | y | I2 | VL | L | N | A | G |
| G41 | LUA DO | 2310 | KIEN GIANG | MRD | T | RL | I | y | I2 | VL | M | N | A | NG |
| G42 | LUA HON COI | 2313 | KIEN GIANG | MRD | T | RL | I | y | I2 | VL | M | M | B | NG |
| G43 | THANH TUA | 2315 | KIEN GIANG | MRD | T | RL | I | y | I2 | VL | M | N | A | NG |
| G44 | MOT BUI | 2332 | KIEN GIANG | MRD | T | RL | I | n |  | VL | L | N | A | NG |
| G45 | NEP CUC | 2367 | NINH BINH | RRD | T | RL | J | y | J4 | E | S | L | C | G |
| G46 | NEP BA LAO | 2368 | NAM DINH | RRD | T | MG | J | y | J4 | E | M | M | C | G |
| G47 | NEP ONG LAO | 2369 | NAM DINH | RRD | T | MG | J | y | J4 | E | M | M | B | G |
| G48 | LUA NGOI | 2371 | NAM DINH | RRD | T | MG | J | y | J4 | E | S | M | C | NG |
| G49 | DT10 | 2395 | u | u | I | u | I | y | I1 | M | L | M | A | NG |
| G5 | NHONG TRANG HAI PHONG | 149 | HAIPHONG | RRD | T | u | I | y | I4 | L | M | N | A | NG |
| G50 | LUA NEP BA THANG DANG 1 | 3323 | QUANG NAM | SCC | T | UP | J | y | J2 | M | M | L | C | G |
| G51 | BA TRANG HUONG | 3332 | QUANG NAM | SCC | T | UP | I | y | I5 | M | S | N | B | NG |
| G52 | BA TRANG HUONG | 3334 | QUANG NAM | SCC | T | UP | I | y | I5 | M | M | N | B | NG |
| G53 | LUA CAN DO | 3351 | u | u | T | u | I | y | I6 | E | M | M | B | NG |
| G54 | LUA LOC DO | 3360 | QUANG NAM | SCC | T | RL | I | y | I6 | M | M | M | B | NG |
| G56 | LUA MAN | 3363 | QUANG NAM | SCC | T | RL | I | y | I5 | M | S | M | B | NG |
| G57 | NEP GHIM HUONG | 3364 | QUANG NAM | SCC | T | RL | I | y | I6 | M | L | M | B | G |
| G58 | NEP HUONG LANG | 3368 | QUANG NAM DA NANG | SCC | T | MG | I | y | I6 | M | S | M | C | G |
| G59 | NEP MAM | 3371 | QUANG NAM DA NANG | SCC | T | u | I | y | I6 | L | L | M | B | G |
| G6 | SOM GIAI HUNG YEN | 170 | u | u | T | u | I | y | I4 | M | L | M | B | NG |
| G60 | NEP CAU | 3400 | QUANG BINH | NCC | T | RL | J | n |  | M | S | M | C | G |
| G61 | NEP RAN | 3402 | QUANG BINH | NCC | T | u | J | y | J2 | E | S | L | C | G |
| G62 | QUANG TRANG | 3426 | QUANG TRI | NCC | T | IR | I | y | I5 | M | S | M | C | NG |
| G63 | CHIEM DO | 3429 | QUANG TRI | NCC | T | u | I | y | I4 | VE | M | M | B | NG |
| G64 | VEN DO | 3433 | QUANG TRI | NCC | T | IR | I | y | I5 | M | S | N | B | NG |
| G65 | NUOC MAN DANG 1 | 3443 | QUANG TRI | NCC | T | RL | I | y | I5 | M | M | M | B | NG |
| G66 | LUA MUOI | 3483 | QUANG NGAI | SCC | T | RL | I | n |  | L | M | M | B | NG |
| G67 | LUA TRI DO DANG 2 | 3485 | BINH DINH | SCC | T | RL | I | y | Im | L | S | M | C | NG |
| G68 | NEP 3 THANG | 3487 | BINH DINH | SCC | T | IR | m | y | m | M | S | M | C | G |
| G69 | COC MOI DANG 1 | 3488 | BINH DINH | SCC | T | RL | I | y | I6 | L | M | M | B | NG |
| G7 | TE TRANG HOA BINH | 172 | HOA BINH | NW | T | u | I | y | I4 | M | M | N | B | NG |
| G70 | COC MOI DANG 2 | 3489 | BINH DINH | SCC | T | RL | I | y | I6 | L | M | M | B | NG |
| G71 | LUA THOM | 3493 | BINH DINH | SCC | T | RL | I | n |  | VL | M | M | C | NG |
| G72 | LUA CANG DANG 1 | 3494 | BINH DINH | SCC | T | RL | I | y | Im | M | M | M | C | NG |
| G73 | LUA CANG DANG 1 | 3495 | BINH DINH | SCC | T | RL | I | y | I6 | L | M | M | C | NG |
| G74 | NEP QUA CO RAU DANG 2 | 3497 | BINH DINH | SCC | T | RL | I | y | I6 | M | M | M | C | NG |
| G75 | NEP QUA | 3498 | BINH DINH | SCC | T | RL | I | n |  | L | L | M | A | G |
| G77 | CANG KIEN DANG 1 | 3506 | BINH DINH | SCC | T | RL | I | y | I6 | L | M | M | C | NG |
| G78 | CANG KIEN DANG 2 | 3507 | BINH DINH | SCC | T | RL | I | y | I6 | L | M | M | C | NG |
| G79 | LUA DA DANG 2 | 3508 | BINH DINH | SCC | T | RL | I | y | I6 | M | S | M | C | NG |
| G8 | CHON TU 502 HOC VIEN | 175 | u | u | T | u | I | y | I4 | M | M | N | B | NG |
| G80 | BA KTONG | 3517 | QUANG NGAI | SCC | T | UP | J | y | J3 | L | VL | M | A | NG |
| G81 | BA DO DANG 1 | 3519 | QUANG NGAI | SCC | T | UP | m | y | m | M | M | M | B | NG |
| G82 | BA DU DANG 2 | u | QUANG NGAI | SCC | T | UP | J | n |  | M | L | L | C | na |
| G83 | NEP VANG | 3522 | QUANG NGAI | SCC | T | UP | J | y | Jm | L | L | L | C | G |
| G84 | BA CHO KTE | 3525 | BINH DINH | SCC | T | u | J | y | J3 | M | L | L | B | NG |
| G85 | CHANH CHUI | 3550 | THANH HOA | NCC | T | MG | J | y | J4 | E | M | M | B | NG |
| G86 | TAN NGAN | 3588 | YEN BAI | NE | T | u | J | y | J2 | M | S | L | C | G |
| G87 | KHAU PAN PUA | 3886 | u | u | T | u | J | y | J1 | E | L | L | C | G |
| G88 | BLE MA MUA | 3895 | u | u | T | u | J | y | J1 | E | VL | L | B | NG |
| G89 | KHAU BO KHA | 3947 | u | u | T | u | J | y | J1 | M | VL | L | B | G |
| G9 | LOC TRANG SOM PLAY CAU | 200 | u | u | T | u | I | y | I4 | M | M | N | B | NG |
| G90 | BLAO CLIA | 4812 | HOA BINH | NW | T | UP | J | y | J1 | M | L | L | B | G |
| G91 | BLAO CO KEN | 4815 | HOA BINH | NW | T | UP | J | y | J1 | M | L | L | B | NG |
| G92 | BLAO CO CAM | 4820 | HOA BINH | NW | T | UP | J | y | J1 | M | L | L | C | G |
| G93 | PO LE PO LAU XA | 5034 | NGHE AN | NCC | T | UP | I | y | I5 | E | L | L | B | G |
| G94 | LUA DO | 5111 | THUA THIEN-HUE | NCC | T | UP | I | y | I5 | M | M | M | B | NG |
| G95 | LUA CHAM | 5127 | NAM DINH | RRD | T | RL | I | y | I4 | M | L | M | B | NG |
| G96 | CHIEM RONG | 6191 | NAM DINH | RRD | T | IR | I | y | I5 | M | M | N | A | NG |
| G97 | TAM THOM | 6199 | NAM DINH | RRD | T | IR | m | n |  | M | S | N | B | NG |
| G98 | NGOI TIA | 6203 | NAM DINH | RRD | T | RL | J | y | J4 | VE | S | M | C | NG |
| G99 | LUA CHAM BIEN | 6234 | NINH BINH | RRD | T | RL | I | y | I4 | M | L | M | B | NG |

u = unknown; na = not analyzed; Zone: NE = Northeast; NW = Northwest; RRD = Red River Delta; NCC = North Central Coast; SSC = South Central Coast; CH = Central Highands; SE = Southeast; MRD = Mekong River Delta; Type: T = traditional, I = improved; Ecosystem: IR = irrigated, RL = rainfed lowland; MG = mangrove; UP = upland; DArT P: population assignments based on Structure results using the DArT markers; GBS: accession genotyped by GBS (y) or not (n); GBS P: population assignments based on Structure results using the GBS markers; Maturity class: E = early, M = medium, L= late; Grain length (L): S = short, M = medium, L = long; Grain width (W): N = narrow, M = medium, L = large; Grain type: G = glutinous; NG = non-glutinous

Table S2: List of the reference accessions included in the analyses

|  |  |  |  |  |  |  | Structure | |
| --- | --- | --- | --- | --- | --- | --- | --- | --- |
| ID | Name | Genebank no | Country | Source | Ecos. | EG | DArT | GBS |
| G224 | APO | ORYTAGE_1 | PHILIPPINES | Cirad CC | UP | 1 | 1 | n |
| G225 | ASD 1 | IRGC_6267 | INDIA | Cirad CC | IR? | 1 | 1 | n |
| G226 | FANDRAPOTSY 104 | IRGC_10984 | MADAGASCAR | Cirad CC | IR | 1 | 1 | n |
| G228 | IR64 | IRGC_66970 | PHILIPPINES | Cirad CC | IR | 1 | 1 | y |
| G229 | KHAO DAWK MALI 105 | IRGC_27748 | THAILAND | Cirad CC | RL | 1 | 1 | n |
| G230 | MAKALIOKA 34 | IRGC_6087 | MADAGASCAR | Cirad CC | IR | 1 | 1 | n |
| G231 | NONA BOKRA | IRGC_22710 | INDIA | Cirad CC | IR? | 1 | 1 | n |
| G232 | PETA | IRGC_32571 | INDONESIA | Cirad CC | IR | 1 | 1 | n |
| G233 | POKKALI | IRGC_8948 | SRI LANKA | Cirad CC | IR | 1 | 1 | n |
| G237 | TAICHUNG NATIVE 1 | IRGC_105 | TAIWAN | Cirad CC | IR | 1 | 1 | n |
| G238 | TE QING | IRGC_81093 | CHINA | Cirad CC | IR | 1 | 1 | n |
| G239 | BARAN BORO | IRGC_27509 | BANGLADESH | Cirad CC | IR | 2 | m | n |
| G240 | BLACK GORA | IRGC_40275 | INDIA | Cirad CC | UP | 2 | 1 | n |
| G241 | DULAR | IRGC_32561 | INDIA | Cirad CC | UP | 2 | 1 | n |
| G242 | KASALATH | IRGC_117617 | INDIA | Cirad CC | UP? | 2 | 1 | n |
| G244 | BASMATI 1 | IRGC_27798 | PAKISTAN | Cirad CC | IR | 5 | m | n |
| G246 | DOM SOFID | IRGC_12880 | IRAN | Cirad CC | IR | 5 | m | n |
| G247 | KAUKKYI ANI | IRGC_33188 | MYANMAR | Cirad CC | RL? | 5 | m | n |
| G248 | PANKHARI 203 | IRGC_5999 | INDIA | Cirad CC | IR? | 5 | m | n |
| G249 | AZUCENA | IRGC_328 | PHILIPPINES | Cirad CC | UP | 6 | 2 | y |
| G251 | CHUAN 4 | IRGC_17052 | TAIWAN | Cirad CC | IR? | 6 | 2 | n |
| G252 | IRAT 216 | CIRAD | COTE D'IVOIRE | Cirad CC | UP | 6 | 2 | n |
| G253 | GIZA 171 | IRGC_50750 | EGYPT | Cirad CC | IR | 6 | 2 | n |
| G255 | IAC 165 | GERVEX_8508 | BRAZIL | Cirad CC | UP | 6 | 2 | n |
| G256 | IGUAPE CATETO | IRGC_4122 | BRAZIL | Cirad CC | UP | 6 | 2 | n |
| G257 | IRAT 13 | IRGC_28508 | CÔTE D'IVOIRE | Cirad CC | UP | 6 | 2 | n |
| G258 | KHAO DAM | IRGC_23385 | LAO | Cirad CC | UP | 6 | 2 | n |
| G259 | KINANDANG PATONG | IRGC_23364 | PHILIPPINES | Cirad CC | UP | 6 | 2 | n |
| G260 | M 202 | IRGC_77142 | USA | Cirad CC | IR | 6 | 2 | n |
| G261 | MOROBEREKAN | IRGC_12048 | GUINEA | Cirad CC | UP | 6 | 2 | n |
| G262 | NIPPONBARE | IRGC_12731 | JAPAN | Cirad CC | IR | 6 | 2 | y |
| G263 | ARIETE | EURIGEN_97 | ITALY | Cirad CC | IR | 6 | 2 | n |
| G265 | CG14 | IRD | SENEGAL | Cirad CC | IR | Og | m | n |
| G266 | SO 1 | u | u | AGI | u | u | 1 | n |
| G267 | HCL | u | u | AGI | u | u | 1 | n |
| G268 | KDS | u | u | AGI | u | u | 1 | n |
| G269 | RD6 | u | THAILAND | AGI | RL | u | 1 | n |
| G270 | XNX | u | u | AGI | u | u | 1 | n |
| G271 | FL | u | u | AGI | u | u | 2 | n |
| G272 | SP | u | u | AGI | u | u | 1 | n |
| G273 | SO 3 | u | u | AGI | u | u | 1 | n |
| G276 | SM | u | u | AGI | u | u | 1 | n |
| G277 | RRHD | u | INDIA | AGI | u | u | 1 | n |
| G279 | LE HUONG 9520 | u | u | AGI | u | u | 1 | n |
| G280 | VLD HAND 64 | u | u | AGI | u | u | 1 | n |
| G281 | IET 8616 | u | INDIA | AGI | u | u | 1 | n |
| G282 | SANBAIKE | u | CHIU | AGI | u | u | 1 | n |
| G283 | AMERICA | u | LAO PDR? | AGI | u | u | 2 | n |
| G284 | IR 6279 | u | PHILIPPINES | AGI | u | u | 1 | n |
| G287 | RR654 | u | INDIA | AGI | u | u | m | n |
| G288 | MT 125 | u | u | AGI | u | u | 1 | n |
| G290 | IET 17021 | u | INDIA | AGI | u | u | m | n |
| G291 | PD12 | u | INDIA | AGI | IR | u | 1 | n |
| G292 | MTU 7029 | u | INDIA | AGI | IR | u | 1 | n |
| G293 | MTU 1010 | u | INDIA | AGI | IR | u | 1 | n |
| G294 | M26 | u | u | AGI | u | u | 1 | n |

u=unknown; Ecos.= ecosystem; EG = enzymatic group; IR= irrigated, RL = rainfed lowland; MG= mangrove; UP= upland;

Table S3: List of the accessions used to build the DArT library

| ID | Variety name | Country of origin | Varietal group |
| --- | --- | --- | --- |
| 1 | 63-83 | Senegal | *trop jap* |
| 2 | Apo | Philippines | *indica* |
| 3 | Arborio | Itlay | *temp jap* |
| 4 | Azucena | Philippines | *trop jap* |
| 5 | Baldo 363 | Italy | *temp jap* |
| 6 | Bomba | Italy | *temp jap* |
| 7 | Cigalon | France | *temp jap* |
| 8 | Gambiaka | Mali | *indica* |
| 9 | IAC 165 | Brazil | *trop jap* |
| 10 | IR64 | Philippines | *indica* |
| 11 | Kalinga III | India | *indica* |
| 12 | Khao Dam | Thailand | *trop jap* |
| 13 | Khao Dawk Mali 105 | Thailand | *indica* |
| 14 | Koral | Portugal | *temp jap* |
| 15 | Makalioka 34 | Madagascar | *indica* |
| 16 | Miara | Italy | *temp jap* |
| 17 | Moroberekan | Guinea | *trop jap* |
| 18 | Nipponbare | Japan | *temp jap* |
| 19 | Oryzica Llanos 5 | Colombia | *indica* |
| 20 | Ribe Jaune | Italy | *temp jap* |
| 21 | Swarna | India | *indica* |
| 22 | Tequing | China | *indica* |
| 23 | Thaibonnet | USA | *temp jap* |
| 24 | Vandana | India | *indica* |
| 25 | Viale | Spain | *temp jap* |

*trop jap* = *tropical japonica*; *temp jap* = *temperate japonica*
