# Supplementary material for: Characterization of a panel of Vietnamese rice varieties using DArT and SNP markers for association mapping purposes
Source: BMC Plant Biol. 2014 Dec 19;14:371. doi: 10.1186/s12870-014-0371-7 (PMC4279583; doi:10.1186/s12870-014-0371-7)
Supplement: Additional file 2: Figure S1. — Decay of r2 along physical distance [in kb] in the indica panel. R2 was averaged across marker pairs for 25 kb intervals. Figure S2. Decay of r2 along physical distance [in kb] in the japonica panel. R2 was averaged across marker pairs for 25 kb intervals. [file 12870_2014_371_MOESM2_ESM.pptx]

## Slide 1
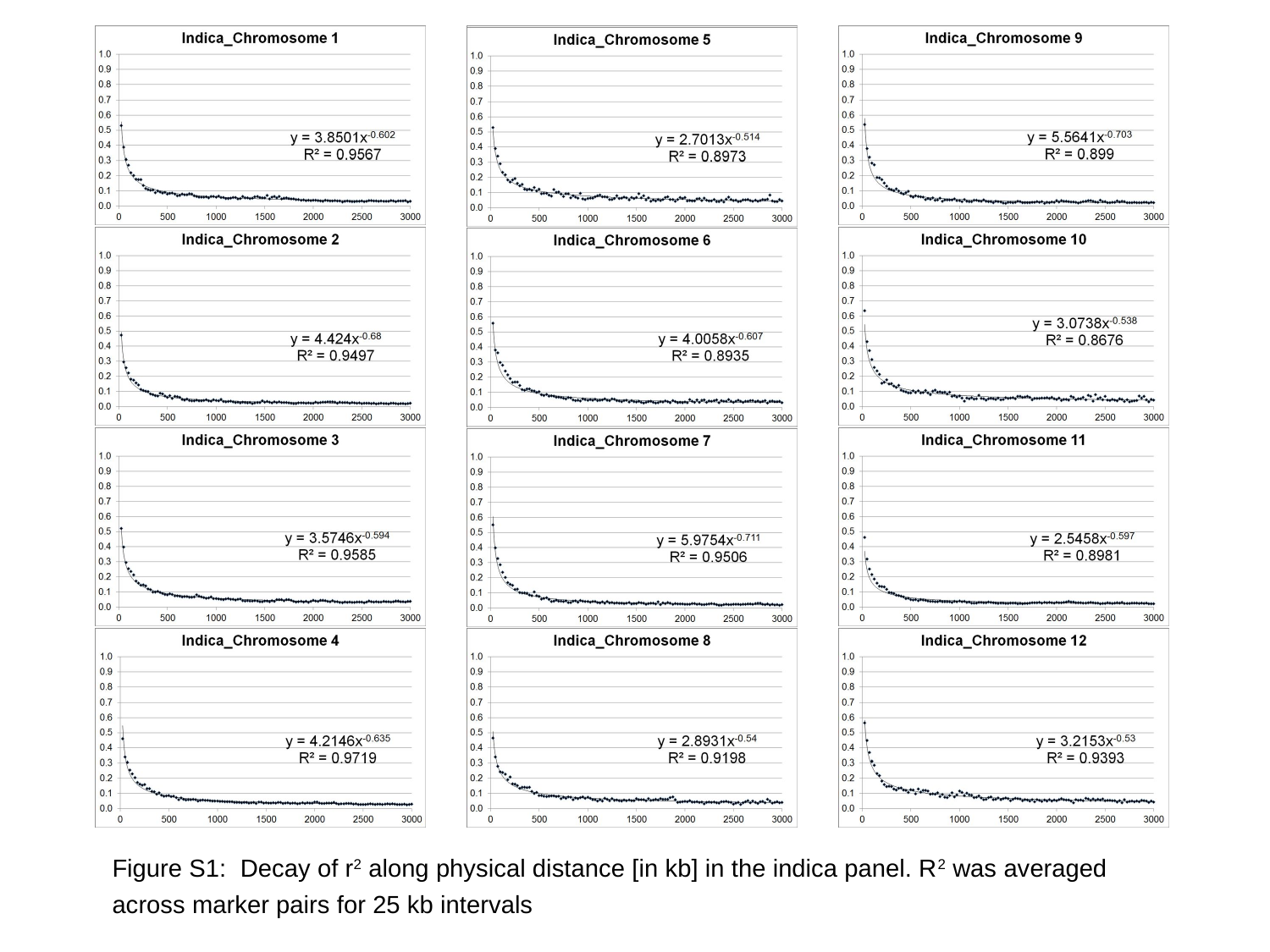

Figure S1: Decay of r2 along physical distance [in kb] in the indica panel. R2 was averaged across marker pairs for 25 kb intervals

## Slide 2
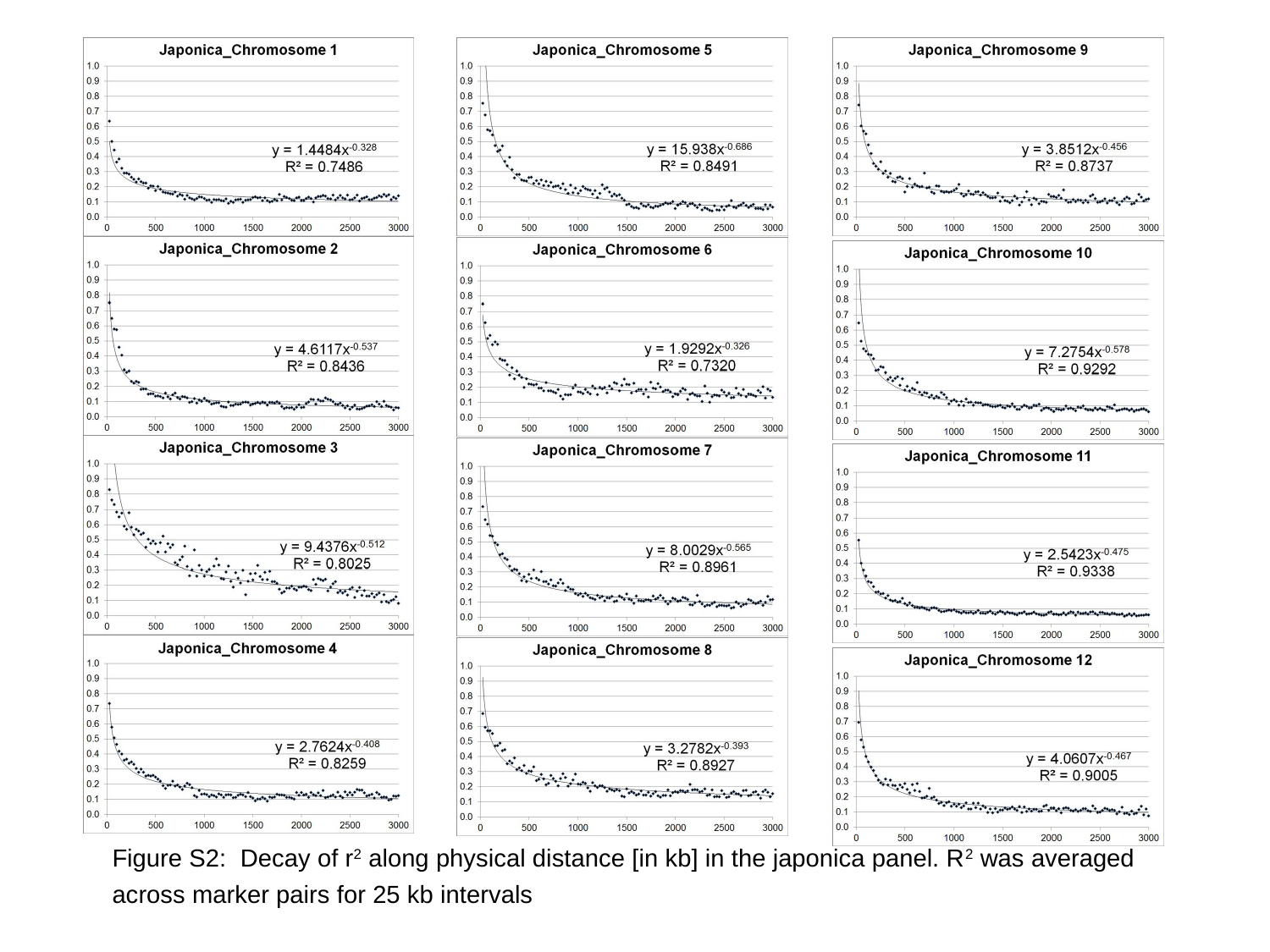

Figure S2: Decay of r2 along physical distance [in kb] in the japonica panel. R2 was averaged across marker pairs for 25 kb intervals
