# Supplementary material for: Characterization of a panel of Vietnamese rice varieties using DArT and SNP markers for association mapping purposes
Source: BMC Plant Biol. 2014 Dec 19;14:371. doi: 10.1186/s12870-014-0371-7 (PMC4279583; doi:10.1186/s12870-014-0371-7)
Supplement: Additional file 3: Figure S3. — Quantile-quantile plots for the full panel (A), the indica panel (B) and the japonica panel (C). [file 12870_2014_371_MOESM3_ESM.pptx]

## Slide 1
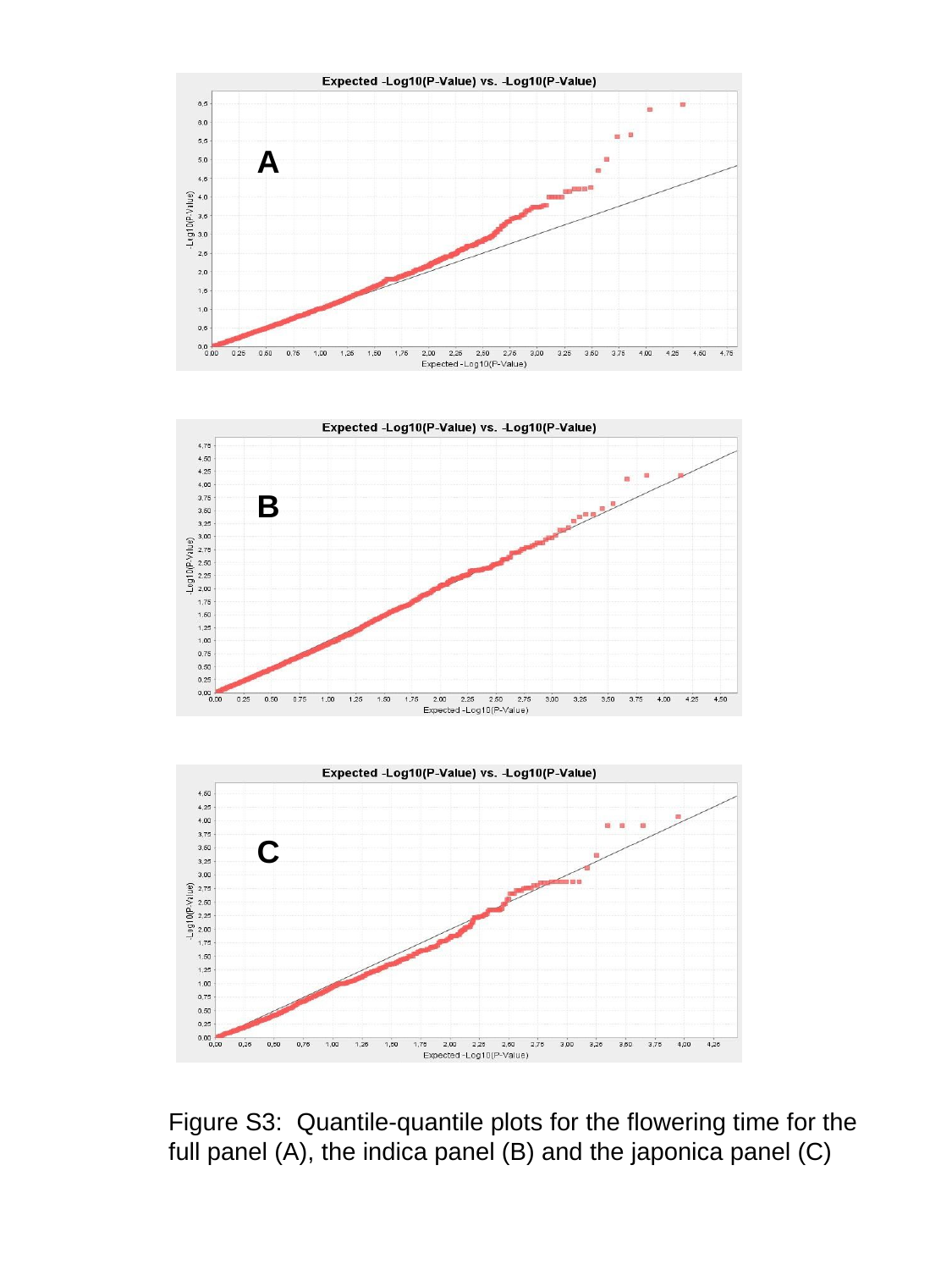

A
B
C
Figure S3: Quantile-quantile plots for the flowering time for the full panel (A), the indica panel (B) and the japonica panel (C)
